# Fucosyltransferase 4-derived peptide bioconjugates on carbon nanotubes enhance antitumor immunity in an ovarian cancer mouse model

**DOI:** 10.3389/fimmu.2026.1821727

**Published:** 2026-05-19

**Authors:** J. J. Guzmán-Mendoza, B. Sánchez-Ramírez, A. Velarde-Calderón, A. M. González-González, C. García-Campuzano, A. Jiménez-Bernal, L. U. López-Bailón, P. Talamás-Rohana

**Affiliations:** 1 Departamento de Infectómica y Patogénesis Molecular. Centro de Investigación y de Estudios Avanzados, Ciudad de México, Mexico; 2 Facultad de Ciencias Químicas, Universidad Autónoma de Chihuahua, Chihuahua, Mexico; 3 Laboratorio de Investigación en Nano y Biomateriales Dentales, Facultad de Estudios Superiores Iztacala, Universidad Nacional Autónoma de México, Ciudad de México, Mexico; 4 Department of Integrative Immunobiology, Duke University School of Medicine, Durham, NC, United States

**Keywords:** antigen-based nanovaccine, carbon nanotubes, CD8 T cell immunity, nanotechnology-based immunotherapy, ovarian cancer, tumor-associated antigen

## Abstract

Ovarian cancer is characterized by a profoundly immunosuppressive tumor microenvironment, which has limited the clinical success of immunotherapy approaches. Antigen-based nanovaccines represent a promising strategy to enhance antitumor immunity by improving antigen delivery and immune activation. In this study, we evaluated carbon nanotubes (CNTs) bioconjugated with a peptide derived from tumor-associated antigen fucosyltransferase 4 (FUT4) as an immunomodulating platform in an immunocompetent ovarian cancer mouse model. FUT4 expression was confirmed in the ID8-Defb29/Vegf-a (ID8DVLuc) ovarian cancer model using flow cytometry and confocal microscopy. C57BL/6 mice were immunized with free peptide combined with a conventional adjuvant (PEP37 Adj), FUT4 peptide–conjugated CNTs (*f*-CNTs), or *f*-CNTs combined with adjuvant (*f*-CNTs Adj), and compared with nonimmunized controls. Immune profiling revealed increased leukocyte infiltration and enrichment of M1-like macrophages, dendritic cells, and CD8^+^ T cells, along with a reduction in regulatory T cells. *f*-CNT immunization also induced enhanced humoral responses, increased splenocyte proliferation upon *ex vivo* peptide restimulation, and increased *ex vivo* CD8^+^ T-cell–mediated cytotoxicity. Exploratory RNA sequencing of ascites-derived cells from *f*-CNT–immunized mice suggested the upregulation of genes associated with antigen processing and presentation, CD8^+^ T-cell activation, and Th1-type immune responses. Immunized mice exhibited reduced ascites accumulation, delayed disease progression, as assessed by bioluminescence imaging, and prolonged survival, with the most pronounced effects observed in the *f*-CNT group. Based on these findings, we propose that FUT4 peptide–conjugated CNTs function as an antigen-based nanoplatform that enhances antitumor immune responses in a murine ovarian cancer model. While additional studies are needed to further validate antigen specificity and therapeutic robustness, this work supports the potential of CNT-based nanocarriers as immune-modulating platforms for ovarian cancer immunotherapy.

## 1 Background

Ovarian cancer remains a challenge in oncology and is the leading cause of gynecological cancer-related mortality worldwide. Despite advancements in surgical techniques and chemotherapeutic regimens, the survival rates for patients diagnosed with advanced ovarian cancer remain poor, with a five-year survival rate of less than 30%, an estimated increase of 3.7% in cases, and 4.7% in deaths in 2020 (1, 2). High recurrence rates and the emergence of chemoresistance underscore the urgent need for novel therapeutic strategies that address the complex biological features of ovarian tumors. In this context, immunotherapy has emerged as a promising approach; however, its effectiveness in ovarian cancer is limited by the profoundly immunosuppressive tumor microenvironment (TME) and the low intrinsic immunogenicity of tumor cells (3, 4), highlighting the need for alternative immunomodulatory strategies.

One emerging approach to overcome these challenges involves the use of nanomaterials as immunomodulatory platforms for the delivery of tumor-associated antigens (5, 6) Among these, carbon nanotubes (CNTs) have attracted considerable attention because of their unique physicochemical properties, including high surface area, structural stability, and versatile functionalization capabilities (7, 8). CNTs have demonstrated biocompatibility and immune-modulatory potential, supporting their use as nanocarrier platforms in cancer immunotherapy (9). Recent studies have shown that CNT-based systems can enhance immune responses by facilitating the delivery of antigens, adjuvants, and therapeutic peptides both *in vitro* and *in vivo (*9–11).

Fucosyltransferase-4 (FUT4), is an α1,3-fucosyltransferase that catalyzes the terminal addition of fucose residues to type II lactosamine chains, generating predominantly Lewis X (LeX/CD15) and, contributing to sialyl-Lewis X (sLe X) structures, and has been implicated in tumor progression and immune evasion mechanisms (12–14). The cancer-related relevance of this aberrant glycosylation pattern is specifically linked to the pathological overexpression of FUT4 in malignant cells, which increases the surface density of LeX/sLeX motifs. This altered glycosylation enhances tumor cell adhesion, migration, and metastatic dissemination (13, 15).

Overexpression of FUT4 increased CD15/CD15s expression and significantly enhanced adhesion of non-small cell lung cancer cells to brain-derived endothelial cells. Importantly, this overexpression also disrupted the endothelial monolayer and promoted a metastatic-like phenotype. Notably, even originally non-metastatic cells acquired metastatic-like behavior upon FUT4 overexpression (13). Overexpression of FUT4 directly altered the migration and invasion capacity of melanoma cells. Mechanistically, these effects were associated with PI3K/Akt pathway activation, supporting a direct signaling link between FUT4-driven glycosylation and metastatic behavior (14).

Importantly, the literature also supports a role in immune escape. FUT4-driven Lewis X-rich glycans can engage C-type lectin receptors expressed by dendritic cells and macrophages, particularly DC-SIGN-related pathways, favoring tolerogenic antigen sensing. Recognition of tumor-associated fucosylated Lewis antigens by these lectin receptors has been associated with increased production of IL-10 and other immunoregulatory mediators, promoting a shift away from effective Th1/cytotoxic antitumor immunity toward a more suppressive or Th2-biased response (15).

FUT4 overexpression has been reported in several malignancies, including acute lymphoblastic leukemia, colon cancer, breast cancer, pancreatic cancer, lung cancer, and gastric cancer (16–19), and has been linked to processes such as epithelial–mesenchymal transition, invasion, proliferation, and metastasis through the regulation of metalloproteinases and multidrug resistance pathways (14, 20). Notably, elevated FUT4 expression is correlated with poor prognosis and enhanced immunosuppressive signaling in lung adenocarcinoma, including associations with ERBB signaling and PD-1 expression (13). These observations support the rationale for exploring FUT4-derived peptides as tumor-associated antigens in immunotherapeutic strategies.

In previous work, we employed bioinformatics approaches to design a multiepitope peptide (PEP37) derived from the FUT4 sequence, which was subsequently bioconjugated onto CNTs via an ester bond, generating FUT4 peptide-bioconjugated CNTs (*f-*CNTs). These nanoconjugates were physicochemically characterized and evaluated for cytotoxicity in the SKOV-3 and J774A.1 cell lines, demonstrating no significant cytotoxic effects. Additionally, *f-*CNTs were shown to activate and polarize macrophages toward an M1-like phenotype *in vitro*, suggesting their potential as immunomodulatory nanocarrier platforms (21).

In the present study, we evaluated the immunomodulatory effects of *f-*CNTs in an immunocompetent murine model of ovarian cancer. Specifically, we assessed their ability to modulate immune cell populations within the tumor-associated microenvironment, enhance antigen-directed immune responses, and influence disease progression and survival. By integrating nanotechnology-based antigen delivery with tumor immunology, this work aims to provide insight into the potential of CNT-based nanoconjugates as immune-modulating platforms for ovarian cancer.

## 2 Methods

### 2.1 Fucosyltransferase 4-Bioconjugated CNTs

FUT4 bioconjugated CNTs were previously synthesized, bioconjugated, and fully characterized as described by Guzman-Mendoza et al. (2024) (21). All immunizations were performed using sterile PBS-dispersed functionalized carbon nanotubes (*f-*CNTs). The formulation remained stably dispersed under physiological conditions and exhibited a ζ-potential of −27.0 ± 0.70 mV, consistent with successful surface functionalization and colloidal stability. Briefly, *f*-CNTs were generated through our previously standardized esterification-based bioconjugation strategy, in which a chemically synthesized 37-amino-acid FUT4-derived peptide corresponding to the sequence SARYKFYLAFENSRHVDYRRYFRWRRSFAVHITSFWS (designated PEP37) was covalently immobilized onto the CNT surface through ester bond formation. This process achieved a peptide loading of 7.77 mg of peptide per 100 mg of *f*-CNTs. The resulting *f*-CNTs had an average length of 0.320 ± 0.129 µm and showed no significant batch-dependent variations among independently prepared formulations.

### 2.2 ID8-Defb29/Vegf-a cell line

C57BL/6 murine ovarian surface tumorigenic epithelial ID8 cells (22) were lentivirally cotransduced with *Vegf-a* and *Defb29* and selected with puromycin and neomycin to accelerate malignant progression upon intraperitoneal injection (23) through the recruitment of tumor-promoting myeloid cells (24). The ID8-Defb29/Vegf-a cell line used in this study was kindly provided by Dr. José R. Conejo-Garcia under a Material Transfer Agreement (MTA) from The Wistar Institute of Anatomy and Biology (Philadelphia, PA, USA). This cell line is derived from the MOSEC ID8 line originally generated at The University of Kansas Medical Center and was transferred in accordance with the terms of the MTA (December 12, 2011) for research purposes.

### 2.3 Animals

Five-week-old C57BL/6 female mice were obtained from the *Unidad de Producción y Experimentación de Animales de Laboratorio* (UPEAL) at CINVESTAV and maintained under standard laboratory conditions with food and water *ad libitum*.

### 2.4 Ovarian cancer model (ID8DVLuc/C57BL/6)

The ascitogenic model of peritoneal carcinomatosis (ID8-Defb29/Vegf-a), which is characterized by a profound accumulation of immunosuppressive myeloid-derived cells within the tumor microenvironment, was used as an ovarian cancer model.

The ID8-Defb29/Vegf-a cell line is a syngeneic murine ovarian cancer model derived from C57BL/6 ovarian surface epithelial cells, genetically modified to overexpress β-defensin 29 (Defb29) and VEGF-A, which together promote enhanced leukocyte recruitment, angiogenesis, rapid peritoneal dissemination, and robust malignant ascites formation. Compared with parental ID8 cells, this variant generates a more aggressive, highly immunosuppressive tumor microenvironment, characterized by profound accumulation of tumor-associated macrophages, myeloid-derived suppressive populations, and regulatory lymphoid components.

This model was specifically selected because it closely recapitulates the advanced peritoneal carcinomatosis and ascites-dominant immune landscape, making it particularly suitable for evaluating the capacity of immune-modulating CNT-based therapeutic nanovaccines to remodel suppressive myeloid populations and restore productive antitumor T-cell responses. The ID8-Defb29/Vegf-a (ID8DVLuc) cell line (1 × 10^6^ cells/mouse) was intraperitoneally inoculated into female C57BL/6 mice.

The mice were randomly assigned to experimental groups. Sample sizes were determined based on prior exploration studies using this model and resource availability for each experiment. The mice were immunized subcutaneously with the antigens. Preimmune serum was collected before immunization. The immunization protocol (described in detail below) was followed, and on day 35, the mice were inoculated with ID8DVLuc cells or PBS (a control without cancer).

Disease progression was monitored using body weight, ascites volume, and bioluminescence imaging, which are commonly employed as surrogate measures in the ID8DVLuc intraperitoneal ovarian cancer model. This model involves the development of disseminated peritoneal implants rather than a single measurable solid tumor mass; therefore, solid tumor nodules were not quantified.

*In vivo* bioluminescence imaging (Newton 7.0, Vilber) was used to monitor tumor development. To induce the luminescent signal, D-Luciferin (PerkinElmer, Cat. No. 122799) was administered intraperitoneally (150 mg/kg). The mice were anesthetized before D-Luciferin injection using a Vilber 7.0 anesthesia system (Newton 7.0, Vilber), which delivered 3% isoflurane in 100% oxygen. Induction occurred within 2 to 3 minutes, at which time a surgical plane of anesthesia was achieved, as indicated by the absence of the pedal withdrawal reflex. Maintenance was sustained at 1.5 to 2% isoflurane throughout the imaging sessions. This protocol was implemented to minimize animal distress during *in vivo* bioluminescence imaging of tumor progression, ensuring that the animals remained unconscious and unresponsive throughout the process. Isoflurane was chosen because of its rapid induction, controllable depth, and minimal interference with luciferase activity, making it ideal for imaging studies. The animals were placed on a heating stage to prevent hypothermia and were continuously monitored for vital signs and anesthetic depth.

**Scheme 1:**
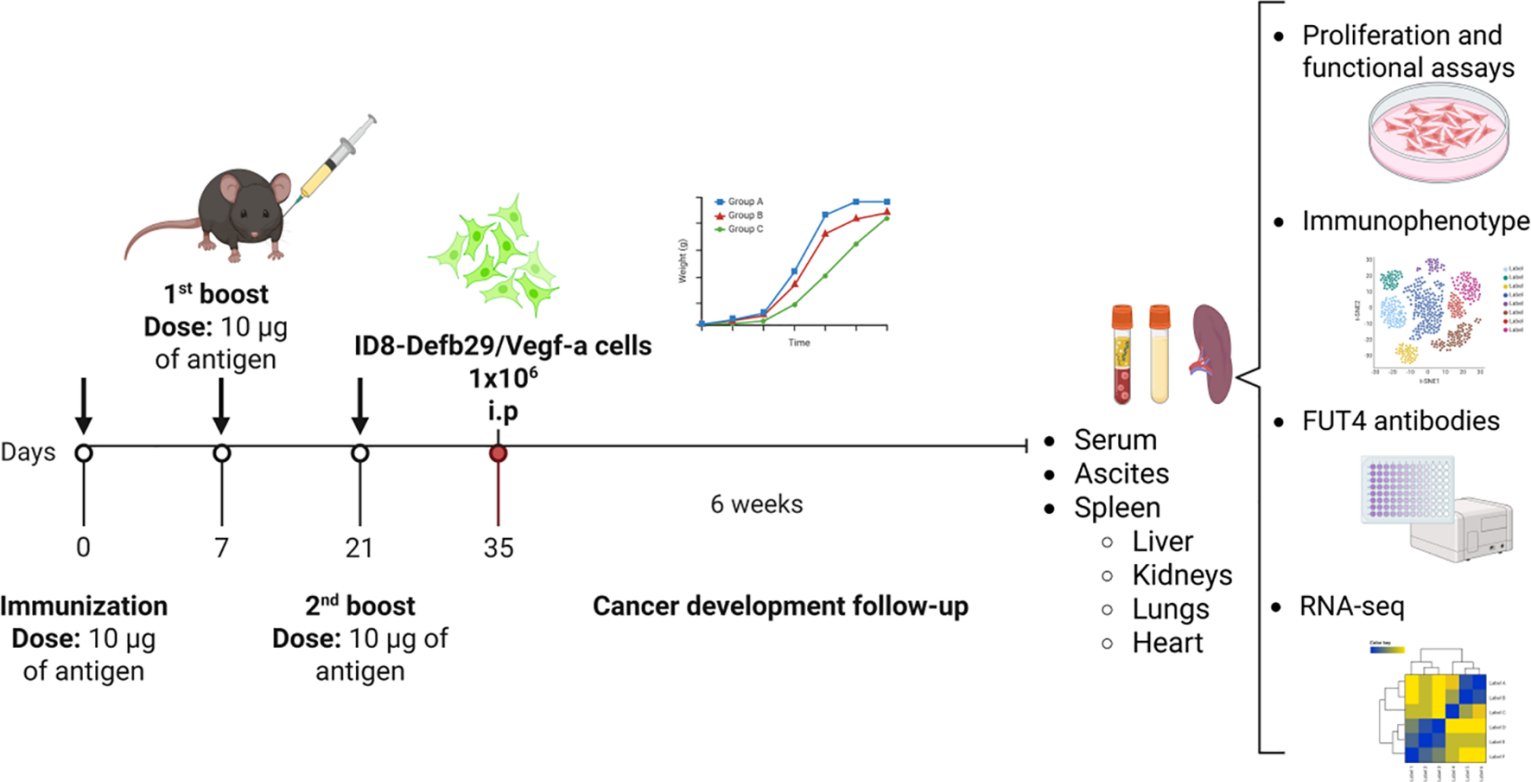
Immunization protocol and experimental design. The experimental groups were immunized with PEP37 plus adjuvant (PEP37 Adj), *f-*CNTs, or *f-*CNTs plus adjuvant (*f*-CNTs Adj). Nonimmunized mice were used as a control for cancer development, and nonimmunized mice without ID8DVLuc cells were used as a basal control. Created in BioRender. Velarde, A. (2026) https://BioRender.com/5ujr3zz.

The humane endpoint was set at a weight increase of more than 30 g, as this model induces ascitic fluid accumulation with a sudden weight increase. For survival studies, the mice were monitored until the endpoint. The mice were euthanized via intraperitoneal injection of sodium pentobarbital at a dosage of 200 mg/kg. This method induces rapid, deep anesthesia followed by respiratory and cardiac arrest. The absence of reflexes and responses to stimuli was confirmed before cervical dislocation was performed as a secondary physical method to ensure death. This protocol guarantees a humane endpoint and complies with all relevant ethical standards. Retroorbital venous blood samples were collected from anesthetized mice. Subsequently, the ascitic fluid was aseptically extracted by percutaneous puncture of the abdominal cavity using a sterile syringe. Finally, animals were sacrificed by cervical dislocation, and the spleen was also aseptically extracted. Spleen cells were isolated by centrifugation at 1500 rpm for 5 min at 4 °C. The resulting cell pellet was collected for further immunophenotyping analyses. The liver, heart, kidneys, and lungs were used for histological analysis (**Scheme 1**).

### 2.5 Immunization protocol

Five-week-old female C57BL/6 mice were used as the experimental model to evaluate immune responses, which was considered the ovarian cancer model. The mice were randomly assigned to the experimental groups, which included the PBS control, PEP37 plus adjuvant, *f-*CNTs, and *f-*CNTs plus adjuvant groups (Titer gold max, Sigma–Aldrich, Cat No. T2684). Nonimmunized mice with or without inoculation of ID8DVLuc cells served as controls. Animals were immunized subcutaneously (s.c.) with different antigens (PBS, PEP37 plus adjuvant, *f-*CNTs, or *f-*CNTs plus adjuvant) (**Scheme 1**).

Venous blood samples were collected via the tail vein before immunization to obtain preimmune serum. The first immunization (day 0) contained 10 µg of the antigen, followed by two booster immunizations on days 7 and 21 with the same antigen concentration.

All procedures were approved by the Institutional Animal Care and Use Committee (UPEAL) of the Centro de Investigación y de Estudios Avanzados (Cinvestav), complying with NOM-062-ZOO-1999 guidelines (project identification number: 0223-17).

### 2.6 Fucosyltransferase 4 expression assay in ID8DVLuc cells

The ID8DVLuc cell line was cultured in high-glucose DMEM (Sigma–Aldrich, Cat. No. D6429) supplemented with 5% FBS (Corning, Cat. No. 35–010) and 1% insulin, transferrin, and sodium selenite (ITS) (Sigma–Aldrich, Cat. No. I3146) at 37 °C in 5% CO_2_. The cells were used within 10 passages to maintain quality.

FUT4 presence in the ID8DVLuc cell line was evaluated under different experimental conditions to characterize its detection profile. FUT4 was assessed at both the mRNA and protein levels. For PCR analysis, the following primers were used: forward 5′-GGATGAACTTCGAATCGCCC-3′ and reverse 5′-AGCTGGTGGTAGTAACGGAC-3′ (T4 Oligo). For protein detection, Western blot analysis was performed using an anti-FUT4 rabbit polyclonal antibody (ABclonal, Cat. No. A16320) at a 1:1000 dilution. Additionally, confocal microscopy and flow cytometry were employed to evaluate FUT4 protein localization and expression under different conditions. In particular, flow cytometry was used to quantify relative FUT4 protein expression based on mean fluorescence intensity (MFI). ID8DVLuc cells were analyzed under basal conditions, after stimulation with murine ascitic fluid, and following recovery from the ascitic fluid of mice six weeks after inoculation.

### 2.7 Serum antibody detection

Peripheral blood was centrifuged to recover immune serum, and FUT4-reactive antibody isotypes (IgG1, IgG2a, IgG2b, IgA, and IgM) were quantified via ELISA. ID8DVLuc protein extracts served as the FUT4 expression control. The plates were coated overnight at 4 °C with 10 µg/mL extract in 0.05 M sodium carbonate buffer (pH 9.6). After blocking, the serum samples (1:100) were incubated overnight, followed by incubation with HRP-conjugated secondary antibodies for each isotype (1:1000). ABTS substrate was added, and absorbance at 405 nm was measured using a Multiskan FC plate reader (Thermo Scientific).

### 2.8 Immune characterization

Spleens and cells from ascitic fluid or peritoneal lavage were used for immunophenotyping, *in vitro* proliferation assays, *ex vivo* cytotoxicity, and total RNA isolation.

For immunophenotyping, multiparametric flow cytometry was performed via a Sysmex XF-1600 system flow cytometer. Two panels were employed: one for lymphoid cells and the other for myeloid cells. Cell viability was assessed with a Zombie Violet™ Fixable Viability Kit (BioLegend, Cat. 423113). The antibodies used were summarized in **Supplementary Table 1**. Fluorescence minus one (FMO) controls and cells individually stained with each fluorochrome were used to perform channel compensation. All data were analyzed using Kaluza 2.2 software (Beckman Coulter, USA), and dimensionality reduction analysis was conducted via the t-SNE algorithm in FlowJo v10.9.0 (Becton, Dickinson, USA).

### 2.9 Proliferation assay

Splenocyte proliferation in response to *ex vivo* peptide restimulation was evaluated. Splenocytes were cultured at 3 × 10^6^ cells/well in RPMI-1640 medium supplemented with 10% FBS, 1% penicillin/streptomycin, 2 mM glutamine, 1 mM sodium pyruvate, 1% nonessential amino acids, and 50 µM β-mercaptoethanol. The cells were stimulated *ex vivo* with 0.5 µg of PEP37 antigen for 72 h. Proliferation was assessed via a Click-iT EdU Kit (Invitrogen, Cat. C10337) following the manufacturer’s protocol. ConA-stimulated and unstimulated splenocytes from healthy mice were used as positive and negative controls, respectively. The stained cells were analyzed on a BD LSRFortessa™ cytometer.

### 2.10 *Ex vivo* cytotoxicity assay

The cytotoxic activity of splenocytes isolated from immunized mice against ID8DVLuc tumor cells was analyzed via coculture as described by Pimentel et al. (2020) (25). Luminescence measurements were performed to evaluate tumor cell viability, and flow cytometry was used to quantify CD8^+^ intracellular cytokine production. Tumor cell viability was assessed via a luciferase-based luminescence assay. The wells were subsequently washed twice with PBS (1 mL), after which 150 μL of 1× passive lysis buffer (PLB) was added to each well. The plates were placed on a shaker at 300 rpm for 20 min at RT. The lysates (50 μL) were transferred to a 96-well plate, D-luciferin substrate (50 μL) was added, and luminescence was measured in a Fluoroscan Ascent FL (Thermo-Scientific). Supernatants containing floating effector cells were collected for flow cytometry staining. Effector cell activation was analyzed via flow cytometry. The collected cells were stained with anti-CD8, anti-GrB, anti-IFNγ, and anti-TNF antibodies. Brefeldin A was added to detect intracellular cytokines, and the cells were incubated for 2 h at 37 °C with 5% CO_2_ before staining was complete. Relative luminescence units (RLUs) were normalized to those of control wells containing ID8DVLuc tumor cells alone. Flow cytometry data were used to analyze cytokine production and effector cell activation.

### 2.11 RNA isolation and RNA sequencing

Ascitic fluid cells were isolated by centrifugation at 1500 rpm for 5 min at 4 °C, as described previously. The resulting cell pellet from each mouse was collected, and total RNA was extracted independently via a RNeasy Mini Kit (Cat. 74104; Qiagen). Following the provider’s protocol, RNA integrity and purity were monitored. The RNA was further cleaned with a DNase I amplification-grade kit (Cat. 18068015; Invitrogen). Purified RNA from 4 mice per condition was pooled, and RNA sequencing was performed as an exploratory, hypothesis-generating approach to identify immune-related transcriptional programs associated with *f*-CNT immunization.

Library construction (TruSeq-stranded mRNA) was assessed via the NovaSeqX platform and RNA sequencing. The reference used was *Mus musculus* (mm10, NCBI_108), the type of paired-end reads was 151, and the read length was 151. The type of sequencer used was the Illumina platform. Mapping, expression profile, DEG (read count), and functional annotation (GO) analyses were performed. All quality control, processing, library construction, sequencing, and data analysis procedures were performed by Macrogen, Inc. (Republic of Korea).

DEG data and molecular pathway data were obtained using the DEW Viewer System by Macrogen, Inc. (Republic of Korea) https://degviewer.macrogen.com/.

### 2.12 Histopathological analysis

Histopathological analysis was performed to assess potential tissue alterations associated with exposure to *f*-CNTs as immunogens. The lungs, heart, liver, and kidneys from the immunized animals two weeks after the second booster were analyzed. These organs were chosen because they are important sites for the transformation and/or excretion of xenobiotics. In addition, the lungs and heart were analyzed to verify that there was no accumulation of nanoparticles, since it has been previously reported that these organs can be susceptible to bioaccumulation when they are administered intravenously or inhaled (26, 27). For this purpose, tissue samples were fixed in 4% paraformaldehyde, embedded in paraffin, and stained with hematoxylin and eosin (H&E) according to standard protocols. Slides were examined under optical microscopy for tissue alterations associated with CNT exposure or tissue damage.

## 3 Results

### 3.1 Fucosyltransferase 4 expression is modulated in the ID8DVLuc tumor microenvironment

**Figure 1:**
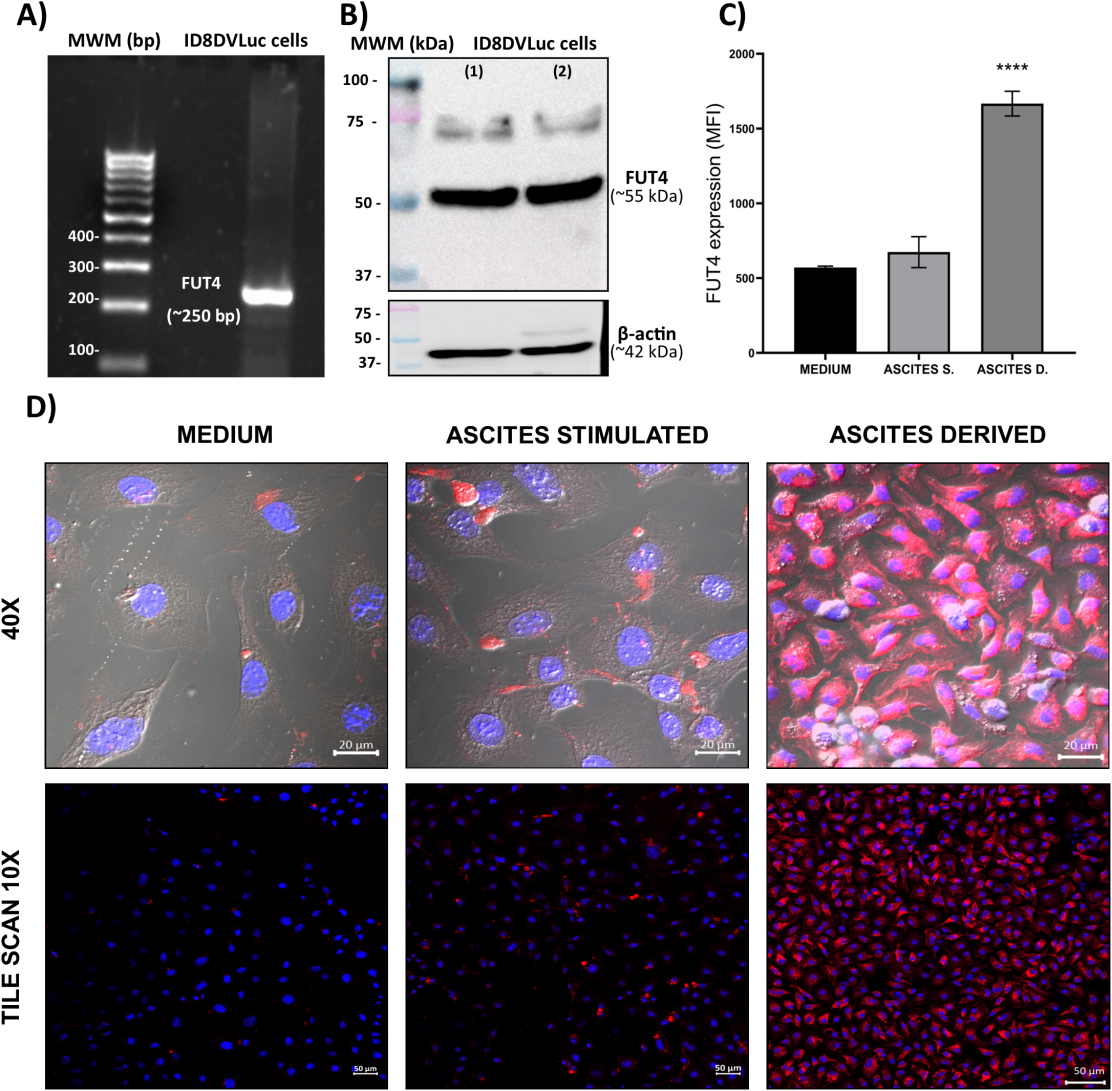
FUT4 is overexpressed in ascites-derived ID8DVLuc cells within the tumor microenvironment. **(A)** detection of FUT4 mRNA by RT-PCR. **(B)** detection of FUT4 protein by western blot analysis (1) medium, (2) ascites stimulated. **(C)** flow cytometry analysis of FUT4 expression, quantified as MFI, mean fluorescence intensity, in non-stimulated ID8DVLuc cells (MEDIUM), cells stimulated with ascitic fluid (ASCITES S), and cells recovered from ascitic fluid (ASCITES D; 6 weeks post-inoculation). **(D)** cell morphology and FUT4 protein localization assessed by immunofluorescence and confocal microscopy. FUT4 is shown in red and nuclei in blue. Data are presented as mean ± S.D. (n = 3, performed in triplicate). Statistical significance was determined by one-way ANOVA followed by Tukey’s multiple comparison test. ****P < 0.0001 vs. non-stimulated control (MEDIUM).

To assess the impact of the tumor microenvironment on FUT4 expression, ID8DVLuc cells were analyzed under *in vitro* conditions and compared with cells exposed to ascitic fluid or recovered from ascites. These conditions represent distinct biological contexts; therefore, the comparison was not intended to establish direct equivalence but rather to evaluate whether environmental factors associated with the ascitic milieu influence FUT4 expression. Under these conditions, changes in FUT4 expression were observed in response to ascites exposure, suggesting that tumor microenvironment–associated signals may modulate target expression in a context-dependent manner. The presence of FUT4 at the mRNA and protein levels was confirmed in the model (**Figures 1A, B**). In addition, to explore whether the TME affects FUT4 protein levels, flow cytometry (mean fluorescence intensity, MFI) was used to quantify relative FUT4 protein expression under different conditions. We evaluated FUT4 expression in ID8DVLuc cells not stimulated, stimulated with ascitic fluid derived from mice, and in cells isolated from ascitic fluid in the same mouse model. The flow cytometry results shown in **Figure 1C** reveal a significant increase in the MFI of FUT4 in cells isolated from ascites compared with that in ascites-stimulated or nonstimulated cells.

In addition, to detect changes in cell morphology and FUT4 expression in stimulated and ascites-derived cells, we performed immunofluorescence (IF) and confocal microscopy. FUT4 expression is shown in red at 40X (**Figure 1D** top row) and in an extended view via the tile scan function (**Figure 1D** bottom row). Microscopic analysis revealed that the morphology of the ascites-derived cells was significantly different, with cells appearing elongated in shape with scant cytoplasm. Additionally, FUT4 is more homogeneously distributed within cells. A tile scan confirmed homogeneous expression in the cells (**Figure 1D** bottom row) and that all cells presented the same phenotype. Together, these results indicate that FUT4 is expressed in the ID8DVLuc model and that its expression profile differs between *in vitro* and ascites-associated conditions. Green fluorescent protein (GFP) expression in ID8DVLuc cells was used as an ID8 marker for IF and flow cytometry (**Supplementary Figure 1**).

### 3.2 *f*-CNT immunization reshapes myeloid and lymphoid immune populations systemically and in the tumor microenvironment

**Figure 2:**
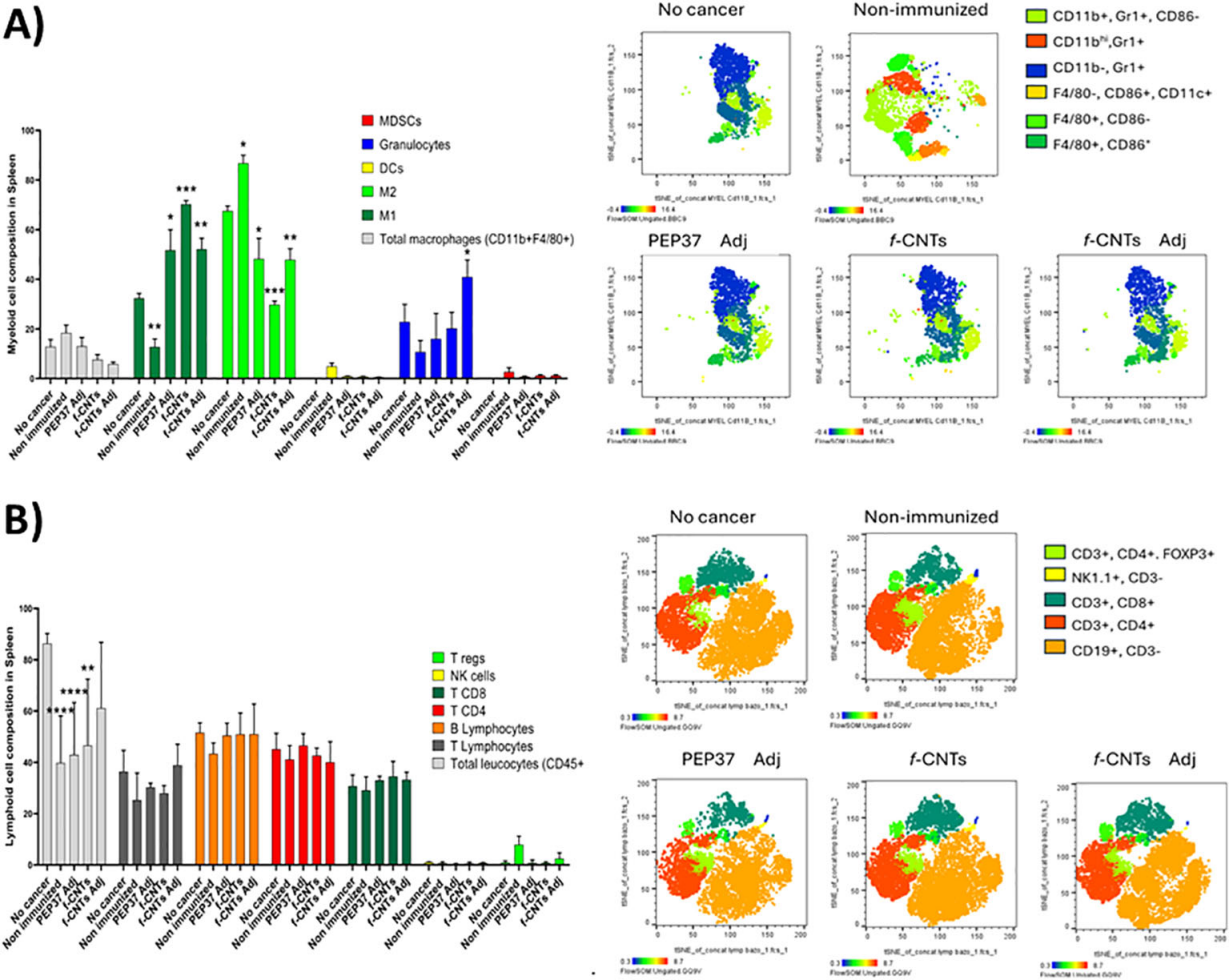
*f-*CNTs immunization remodeled systemic immune populations in the spleen. **(A)** myeloid and **(B)** lymphoid populations were analyzed by flow cytometry. On the left, graphs show the means of frequency of each population across experimental groups (n = 4 mice per group). On the right, dimensionality reduction analysis (t-SNE) illustrates the distribution of immune subpopulations based on the markers used for their identification. Statistical significance was determined by one-way ANOVA followed by Tukey’s multiple comparison test. Asterisks indicate significant differences compared with the non-cancer group (*P < 0.05, **P < 0.01, ***P < 0.001, ***P < 0.0001).

Multiparametric flow cytometry was performed to characterize the immune cells present in the ascitic fluid, as well as to obtain an overview of the systemic environment using spleen cells. The results are presented in two panels (myeloid and lymphoid cells) and two sections, ascites and the spleen (**Supplementary Figure 6**). The results for the spleen cells are shown in **Figure 2**, which provides a panoramic view of the systemic distribution of immune cells, t-distributed stochastic neighbor embedding (t-SNE), a dimensionality reduction algorithm used to visualize high-dimensional flow cytometry data by clustering phenotypically related immune cell populations. The immune population showed a different t-SNE distribution, which was observed in cancer nonimmunized mice compared with that in healthy mice (no cancer), with an increase in mainly myeloid cells (**Figure 2A**). Moreover, in the cancer non-immunized mouse group, we detected an increase in M2-type macrophages, a decrease in M1 macrophages, and an increase in MDSCs, together with a reduction in the proportion of granulocytes; all the above findings are associated with a protumoral profile. In contrast, the immunized groups showed a polarization of macrophages from the M2 to the M1 profile, a decrease in the number of MDSCs, and an increase in the number of granulocytes, which were associated with an antitumor profile. Few changes in the proportions of lymphoid cells were observed, with only a slight decrease in regulatory T (Treg) cells in all immunized groups (**Figure 2B**). Although there were subtle changes in the populations, in general, the same pattern was observed in all immunized groups, with a more significant increase in M1 macrophages in the group immunized with *f-*CNTs, suggesting that these *f-*CNTs enhance M1 polarization in this model.

**Figure 3:**
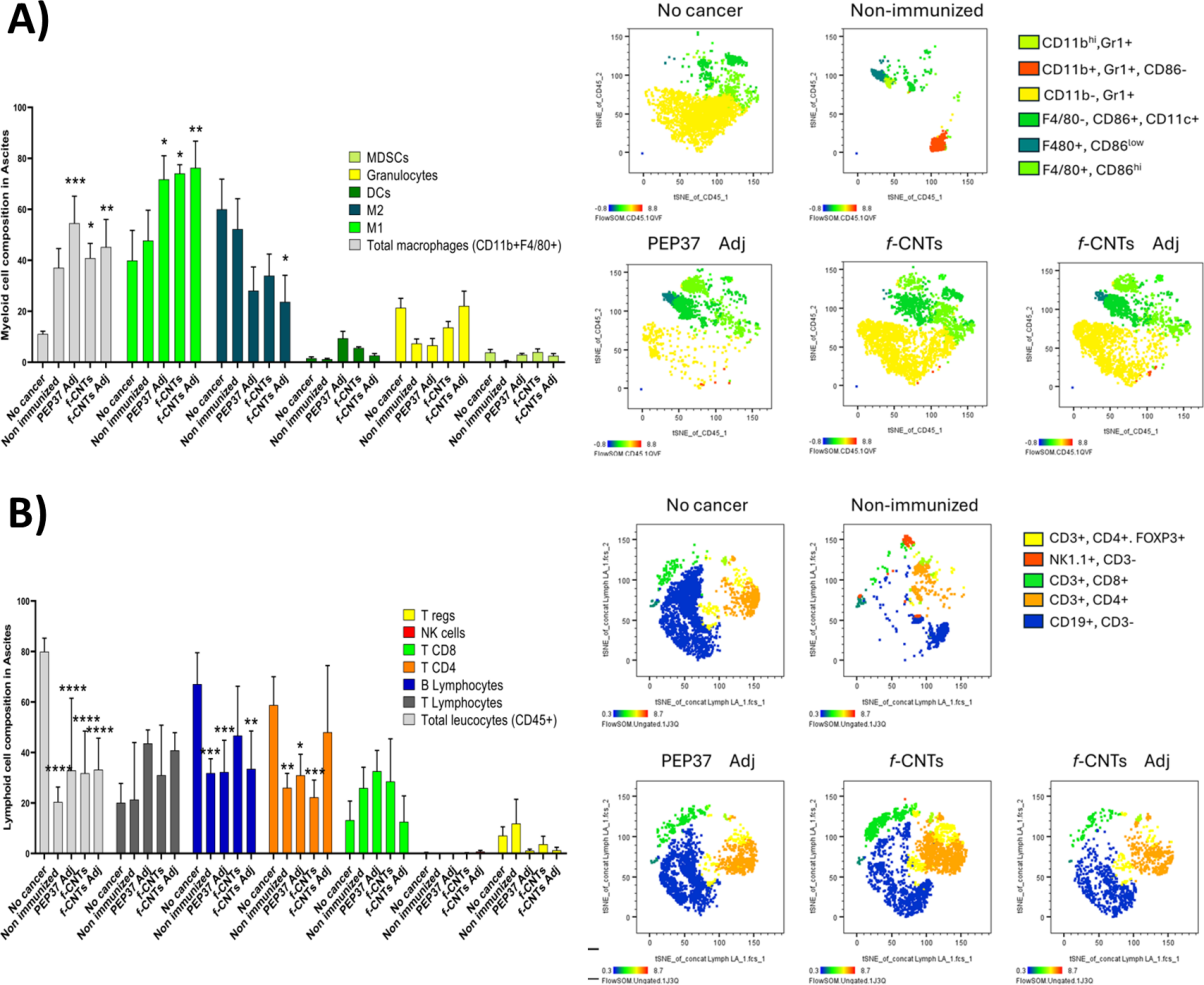
*f-*CNTs immunization reshaped the tumor microenvironment immune landscape in ascitic fluid. **(A)** myeloid and **(B)** lymphoid populations were analyzed by flow cytometry. On the left, graphs show the means of frequency of each population across experimental groups (n = 4 mice per group). On the right, dimensionality reduction analysis (t-SNE) illustrates the distribution of immune subpopulations based on the markers used for their identification. Statistical significance was determined by one-way ANOVA followed by Tukey’s multiple comparison test. Asterisks indicate significant differences compared with the non-cancer group (*P < 0.05, **P < 0.01, ***P < 0.001, ***P < 0.0001).

The distribution of lymphoid and myeloid populations in the ascitic fluid TME is displayed in **Figure 3**. The results obtained were quite similar to those obtained in the spleen in myeloid populations: an increase in the proportion of macrophages and dendritic cells in all immunized groups; a switch from M2 to M1 in the macrophage populations, which was more evident in the *f-*CNTs + Adj group; and an increase in the number of granulocytes in the *f-*CNT groups. The total T lymphocyte population increased in all immunized groups, and the CD8 T-cell population increased in the groups immunized with PEP37 Adj and *f-*CNTs, whereas the CD4 T-cell population increased in the group immunized with *f-*CNTs + Adj. These results suggest that the adjuvant shifted the response toward a Th2 profile, with increase CD4+ T cells and decrease in CD8+ T cells. Additionally, a reduction in the proportion of Treg cells was observed in all immunized groups. The number of B lymphocytes increased only in the *f-*CNT group.

To further evaluate T-cell immune balance, the CD4/CD8 ratio was calculated in both splenic and ascitic compartments. Whereas only modest differences were observed in the spleen, the ascitic fluid from *f*-CNT-immunized mice showed the lowest CD4/CD8 ratio (0.78), markedly lower than observed in the other treatment groups and reflecting a relative enrichment of CD8^+^ T cells at the tumor-associated peritoneal site. This compartment-restricted shift suggests that *f*-CNTs immunization preferentially promotes local cytotoxic immune polarization within the tumor microenvironment (**Supplementary Table 2**).

### 3.3 Exploratory RNA sequencing identifies immune-related transcriptional programs associated with *f*-CNT immunization

This analysis is a systems-level complement to the cellular and functional immunologic findings. Differential expression analysis of genes (DEGs) was performed using RNA sequencing to compare non-immunized vs. PEP37 Adj and non-immunized vs. *f-*CNT. We performed an exploratory RNA-seq analysis of all the DEGs, including both upregulated and downregulated genes. From this comprehensive dataset, we selected genes associated with the previously described pathways related to the polarization switch from M2 to M1 macrophages and to neutrophil subtypes (N1 and N2) within the TME (**Tables 1**, **2**).

**Table 1:** Transcript per kilobase million (TPM) values of the most important genes associated with protumoral and antitumoral immune populations in the TME.

Cell type	Gene	Description	Non-immunized	*f-*CNTs	PEP37 Adj
M1 macrophages	Cd80	CD80 antigen	2.8170	23.2399	4.2403
	Cd86	CD86 antigen	8.5891	56.9675	11.4480
M2 macrophages	Arg1	Arginase	955.6223	218.6696	240.4957
	Cd163	CD163 antigen	29.1808	6.7065	9.2134
N2 neutrophils	Mmp9	matrix metallopeptidase 9	72.6723	11.8290	14.9972
N1 neutrophils	Trpm2	transient receptor potential cation channel, subfamily M, member 2	2.6234	6.9784	4.4563
M2/N2	Vegfa	vascular endothelial growth factor A	145.0166	1.1996	43.3987
M2/N2/Th2	Il10	interleukin 10	3.5264	0.5104	3.0188
T Lymphocytes	Cd3e	CD3 antigen, epsilon polypeptide	1.6288	15.2193	7.734
CD8 T cells	Cd8b1	CD8 antigen, beta chain 1	2.6076	5.9131	6.7885
	Prf1	perforin 1 (pore forming protein)	0.1561	1.8742	0.6160
	Gzma	granzyme A	0.5976	5.9054	3.5829
CD4 T cells	Cd4	CD4 antigen	0.8814	5.9886	2.6025
Th1 T cells	Stat4	signal transducer and activator of transcription 4	2.2418	23.7252	7.2800
Th2 T cells	Gata3	GATA binding protein 3	6.8505	2.6843	3.4954
Th9 T cells	Stat6	signal transducer and activator of transcription 6	56.4713	91.0747	61.2127
Th17 T cells	Stat3	signal transducer and activator of transcription 3	128.5286	69.5628	135.4306
	Rorc	RAR-related orphan receptor gamma	51.1281	0.5721	12.2633
CaOv tumor markers	Wt1	Wilms tumor 1 homolog	321.3447	0.1193	279.4064
	Bmp7	bone morphogenetic protein 7	3.8938	0.4040	4.8184
	Cldn4	claudin 4	0.1687	0.206	0.10170
	Foxj2	forkhead box J2	29.1159	15.2000	16.7026

**Table 2:** Functional summary of enriched immune-related biological processes identified in transcriptomic analysis across treatments.

Functional category	Enriched biological processes (GO terms)	Predominantly enriched in	Biological interpretation
T cell–mediated immunity	T cell activation; leukocyte-mediated cytotoxicity	*f*-CNTs	Enhanced activation of adaptive cellular immune responses
CD8^+^ T cell activation	CD8-positive αβ T cell activation	*f*-CNTs	Promotion of cytotoxic T cell responses
Th1 differentiation	Th1 cell differentiation; regulation of adaptive immune response	*f*-CNTs + PEP37 Adj	Skewing toward a Th1-type immune response
Antigen presentation (MHC)	Antigen processing and presentation	*f*-CNTs	Improved antigen presentation capacity
Leukocyte migration	Leukocyte cell–cell adhesion	*f*-CNTs	Increased immune cell recruitment
Immune regulation	Immune response-regulating signaling pathway	PEP37 Adj	Modulation of immune regulatory pathways
Innate immune response	Response to molecule of bacterial origin	PEP37 Adj	Activation of innate immune-related processes
Cell differentiation	Lymphocyte differentiation; hematopoietic progenitor differentiation	*f-*CNTs + PEP37 Adj	Immune cell development and differentiation

We identified 1149 and 2538 upregulated genes and 750 and 2849 downregulated genes in PEP37 Adj- and *f-*CNT-immunized mice, respectively. In addition, 741 upregulated and 493 downregulated genes were shared between the groups. These findings indicate that some processes are shared between PEP37 Adj- and *f-*CNT-immunized mice. The most relevant genes in the *f-*CNT group were *H2-D1*, *H2-K1*, *B2m*, and *Cd74*. In the PEP37 Adj group, only *Cd74*, which is crucial for antigen presentation, was among the most relevant (**Supplementary Figure 2**). These transcriptional changes are consistent with the modulation of pathways related to antigen processing and presentation.

As a complement to immunophenotyping, this exploratory RNA-seq helps us perform a gene ontology analysis to identify some of the processes implicated in the immunomodulation induced by *f-*CNTs and PEP37 Adj.

**Figure 4:**
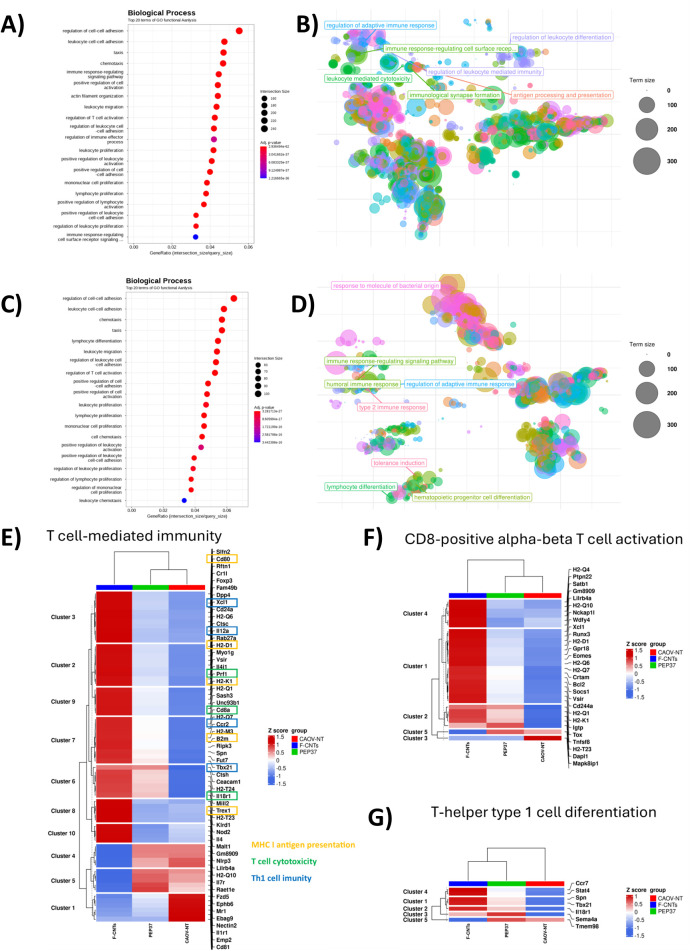
Transcriptomic analysis revealed enrichment of antigen presentation and T-cell–mediated immune pathways following *f-*CNTs immunization. **(A)** The top 20 terms of the GO functional analysis related to biological processes in the *f-*CNT-immunized mice. **(B)** Scatter plot of the most important terms of the GO functional analysis in the *f-*CNT-immunized mice. **(C)** Top 20 terms of the GO functional analysis related to biological processes in PEP37 Adj-immunized mice. **(D)** Scatter plot of the most important terms of the GO functional analysis in PEP37 Adj-immunized mice. **(E)** Top DEGs related to T-cell-mediated immunity. **(F)** Top DEGs related to CD8+ alpha-beta T-cell activation. **(G)** Top DEGs related to T-helper type 1 cell differentiation. The distances between points in the scatter plots represent the similarity between terms, and the circle size is related to the term size.

The biological processes altered in the *f-*CNT-immunized mice are shown in **Figure 4A**. The most important processes were the regulation of cell–cell adhesion, leukocyte–cell adhesion, taxis, chemotaxis, and immune response-regulating signaling pathways (**Supplementary Figure 4**). The PEP37 Adj scatter plot is shown in **Figure 4B**, highlighting the main GO terms. Some interesting GO terms, such as leukocyte-mediated cytotoxicity, immunological synapse formation, antigen processing and presentation, and the regulation of the adaptive immune response, were revealed. On the other hand, the results of the analysis of the PEP37 Adj-immunized mice are shown in **Figures 4C, D**; here, a similar pattern was detected for the regulation of cell–cell adhesion, leukocyte cell–cell adhesion, chemotaxis, taxis, and some differences, such as lymphocyte differentiation, was detected (**Supplementary Figure 5**). In the scatter plot (**Figure 4D**), the processes included the humoral immune response, type 2 immune response, lymphocyte differentiation, and tolerance induction, which is very important because immunization with unconjugated PEP37 Adj cannot be immunogenic enough *per se* to evoke an efficient immune response, resulting in tolerance, which does not occur with the *f*-CNTs. These transcriptional patterns suggest that CNT-based immunization may modulate antitumor immune responses, particularly those associated with cytotoxic T-cell–mediated pathways.

We analyzed three of the most important GO terms in the context of immunological responses. The first is T-cell-mediated immunity; many genes are modulated by PEP37 Adj but in a greater manner by *f-*CNTs. To better understand these genes, we classified them into three categories—MHC-I antigen presentation, T-cell cytotoxicity, and Th1-cell immunity—because these are the most important for generating a robust immune response against cancer cells.

Among the genes involved in MHC-I antigen presentation, Cd80, H2-D1, H2-K1, B2m, and Trex1 were upregulated in *f-*CNTs but to a minor degree in PEP37 Adj-immunized mice (**Figure 4E**). With respect to T-cell cytotoxicity, the upregulated genes were *Prf1*, *Cd8a*, and *Il18r1*; the latter is a marker of T-cytotoxic cells. With respect to Th1 cell immunity, the upregulated genes included *Xcl1*, *Il12a*, *Ccr2*, and *Tbx21*. These genes were more highly upregulated in *f-*CNT-immunized mice than in control mice, except for *Tbx21*, whose expression levels were the same in *f-*CNTs and PEP37 Adj. This information suggests that *f-*CNTs are associated with an anticancer immune response, which could be mediated mainly by T-cytotoxic cells. Thus, we analyzed genes associated with T-cell-mediated cytotoxicity (**Figure 4F**). Most genes were upregulated by *f-*CNT immunization, and some genes in cluster 2 were the same in PEP37 Adj, suggesting that even unconjugated PEP37 Adj can activate the T-cell cytotoxic immune response. In addition, we analyzed the genes involved in Th1 polarization, which is important for generating anticancer immune responses. The genes are displayed in **Figure 4G**. Here, the transcription factors *Stat4* and *Tbx21* were upregulated in PEP37 Adj-immunized mice and highly expressed in *f-*CNT-immunized mice.

The main genes implicated in this process are displayed in **Table 1** as transcripts per kilobase million (TPM) values. These results are consistent with those of flow cytometry immunophenotyping, which revealed an increase in the expression of genes associated with M1 macrophages in the *f-CNT-* and PEP37 Adj-immunized mice but to a lesser degree.

Increases in *Cd80* and *Cd86*, with decreases in *Arg1*, *Cd161*, *Vegfa*, and *Il10*, were observed in the *f-*CNT-immunized mice compared with those in the nonimmunized group. A similar pattern is observed in PEP37 Adj-immunized mice, but at a lower level. With respect to the neutrophil subsets, we observed an increase in *Trmp2*, followed by a decrease in *Mmp9*, *Il10*, and *Vegfa* in *f-*CNTs and a lower grade in PEP37 Adj, similar to the findings for the macrophage subsets. These results suggest that *f-*CNTs induce an antitumoral microenvironment mediated by M1 macrophages and N1 neutrophils, which may help control tumor development and ascitic fluid accumulation.

Additionally, the expression of several genes associated with T lymphocytes increased; among these *Cd3e*, *Cd4*, and *Cd8b1* genes were upregulated, indicating an increase in the number of T lymphocytes in the TME. In addition, the T cytotoxic cells in the TME displayed a functional profile with increases in *Prf1* and *Gzma*, mainly in the *f-*CNT-immunized mice and, at lower levels, in the PEP37 Adj-immunized mice.

Concerning the CD4 subset profile, we analyzed the transcription factors *Stat4*, *Gata3*, *Stat6*, *Stat3*, and *Rorc*, revealing a Th1 and Th9 profile in *f-*CNT-immunized mice. Moreover, the PEP37 Adj-immunized mice displayed a mixed Th1/Th2 profile and a Th17 subset. In addition, the protumoral ovarian cancer genes *Wt1*, *Bmp7*, and *Foxj2* were expressed at low levels in immunized mice, which aligns with the strong antitumoral profile in the TME of *f-*CNT-immunized mice (**Table 1**).

Enriched Gene Ontology (GO) biological processes identified in the transcriptomic analysis (**Figure 4**) were grouped into functional categories to facilitate interpretation in **Table 2**. Processes were assigned to treatment groups based on their relative enrichment patterns, highlighting distinct immune signatures associated with *f-*CNTs and PEP37 Adj immunization.

The multiparametric flow cytometry findings provide cellular-level support for several of the transcriptomic observations, including enhanced M1-like macrophage polarization and increased CD8^+^ effector responses, thereby strengthening the cross-validation between omics-level pathway enrichment and functional immune phenotyping.

### 3.4 Splenocytes from *f*-CNT–immunized mice exhibit antigen-responsive proliferation and CD8^+^ T-cell cytotoxic activity

Previous cytometry results revealed an increase in the T CD8^+^ population and overexpression of genes related to T-cell-mediated cytotoxicity; here, we confirmed that T cytotoxic cells from immunized mice were functional and cytotoxic against ID8DVLuc cells.

**Figure 5:**
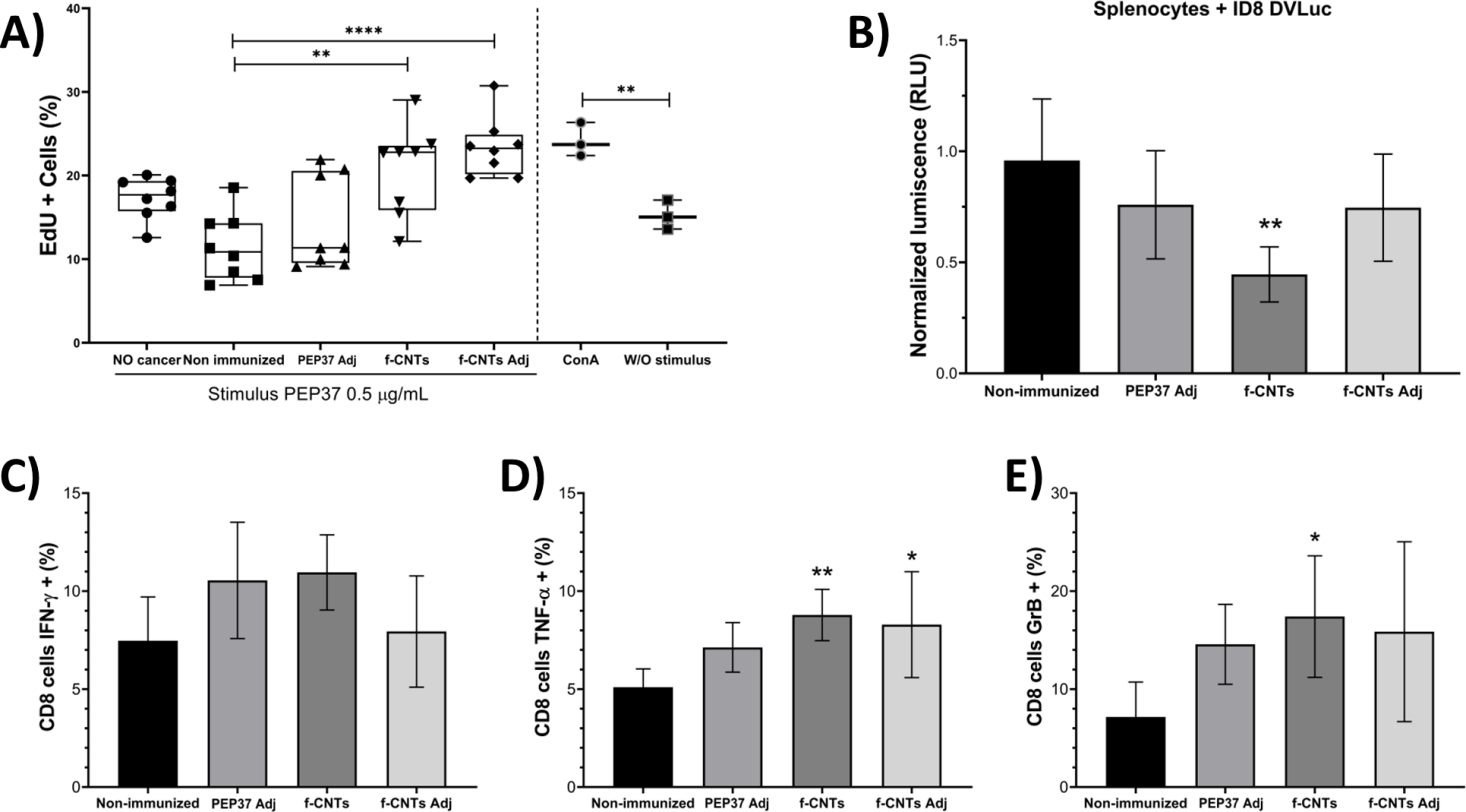
*f-*CNTs immunization enhances antigen-specific proliferation and CD8^+^ T-cell cytotoxic activity. **(A)** proliferation of splenocytes from immunized mice. ConA-stimulated (0.5 µg/mL) and nonstimulated splenocytes from a healthy mouse were used as positive and negative controls. **(B)** *ex vivo* cytotoxicity assay against ID8DVLuc cells. Splenocytes were cocultured with ID8DVLuc cells, and the RLU, luminescence was proportional to the number of viable ID8DVLuc cells. RLU was normalized to 1 relative to nonimmunized mouse splenocytes. **(C)** CD8^+^ cells and IFN-γ^+^ cells. **(D)** CD8^+^ cells, TNF-α^+^ cells. **(E)** CD8^+^ cells, granzyme B^+^ cells. Each bar shows the mean ± S.D. (four mice were used per group, with technical duplicates, n=8). Asterisks indicate significant differences *vs*. the non-immunized group. **** p<0.0001, ** p<0.01, * p<0.05. ANOVA and Tukey’s multiple comparison tests were used.

To analyze whether splenocytes isolated from immunized mice were responsive to the PEP37 antigen, *ex vivo* cell proliferation and cytotoxicity assays were performed. The non-immunized groups did not recognize the antigen, and the cells could not proliferate. In contrast, splenocytes from the immunized groups showed an increase in the proliferative response to PEP37, approximately 15% in PEP37 Adj-immunized mice and 20% in *f-*CNT-immunized mice. Importantly, the response was heterogeneous, since some of the mice exhibited significantly greater proliferation than others did (**Figure 5A**); nevertheless, in the *f-*CNT and *f-*CNT plus adjuvant groups, most of the mice exhibited increased proliferation.

Compared with that in the nonimmunized group, the viability of ID8DVLuc cells in the group in which these cells were cocultured with splenocytes derived from *f-*CNT-immunized mice significantly decreased (**Figure 5B**). In addition, we evaluated CD8+ T cells positive for IFN-γ, TNF-α, and Granzyme B, which are markers of cytotoxicity (**Figures 5C–E**, respectively). Significant differences in TNF-α and Granzyme B production in the *f-CNT-* and *f-*CNT plus adjuvant-immunized groups were observed. These results are in agreement with the RNA-seq results (**Table 1**), as the expression of the *Grza* and *Prf1* genes was lower in the PEP37 Adj-immunized group than in the *f-*CNT-immunized group, and the expression of these genes was greater in the *f-*CNT-immunized group. These results indicate that splenocytes from *f*-CNT–immunized mice exhibit enhanced CD8^+^ T-cell–associated cytotoxic activity against ID8DVLuc cells *ex vivo*.

### 3.5 *f*-CNT immunization induces a sustained humoral response with isotype skewing

**Figure 6:**
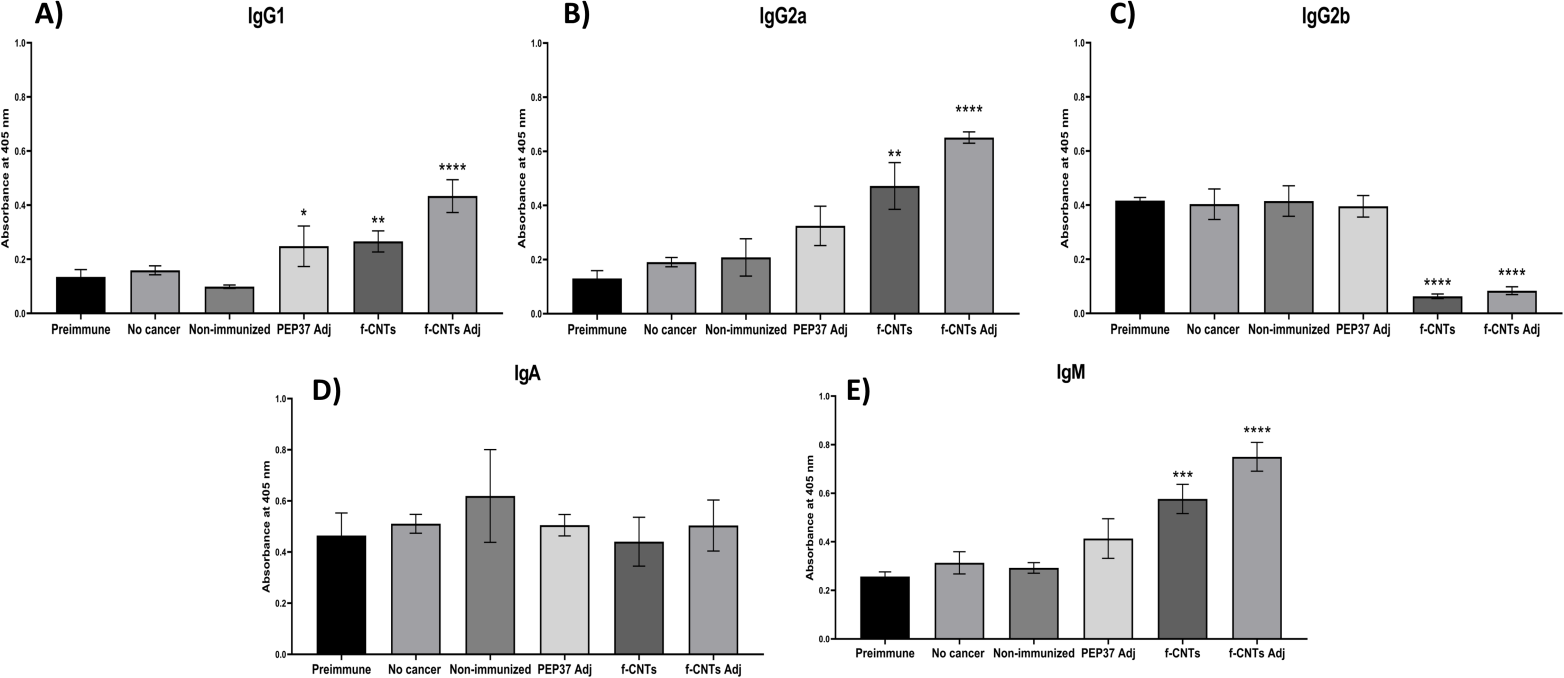
*f-*CNTs immunization induces a Th1-associated humoral response with increased IgG2a and sustained IgM levels. Immunoglobulin quantification of **(A)** IgG1. **(B)** IgG2a. **(C)** IgG2b. **(D)** IgA. **(E)** IgM. Each bar shows the mean ± S.D. Asterisks indicate significant differences *vs*. the non-immunized group **** p<0.0001, *** p<0.001, ** p<0.01, * p<0.05. ANOVA and Tukey’s multiple comparison tests were used. The serum was diluted to 1:100, and the anti-isotype antibodies were diluted to 1:1000.

We quantified IgG1, IgG2a, IgG2b, IgA, and IgM levels in serum collected from immunized mice six weeks after ID8DVLuc inoculation to evaluate the humoral immune response. The results showed that immunization with PEP37 Adj and functionalized carbon nanotubes (*f-*CNTs) elicited a humoral immune response. However, the antibody profiles differed among the groups. Both PEP37 Adj and *f-*CNTs induced comparable levels of IgG1. Notably, the combination of *f-*CNTs with TiterMax adjuvant produced the highest IgG1 titers (**Figure 6A**).

In contrast, IgG2a levels (**Figure 6B**) were significantly increased only in mice immunized with *f-*CNTs alone or *f-*CNTs plus adjuvant, indicating a potential Th1-skewed response in *f-*CNT-immunized mice. Conversely, IgG2b levels were reduced in mice immunized with *f-*CNTs or *f-*CNTs plus adjuvant (**Figure 6C**). No significant differences in IgA production were observed among the groups (**Figure 6D**).

Interestingly, we detected significantly high IgM levels after several weeks in mice immunized with *f-*CNT or *f-*CNT plus adjuvant (**Figure 6E**), revealing strong and constant stimulation by the *f-*CNTs. This did not occur with PEP37 Adj immunization.

### 3.6 *f*-CNT immunization is associated with delayed disease progression and improved survival

**Figure 7:**
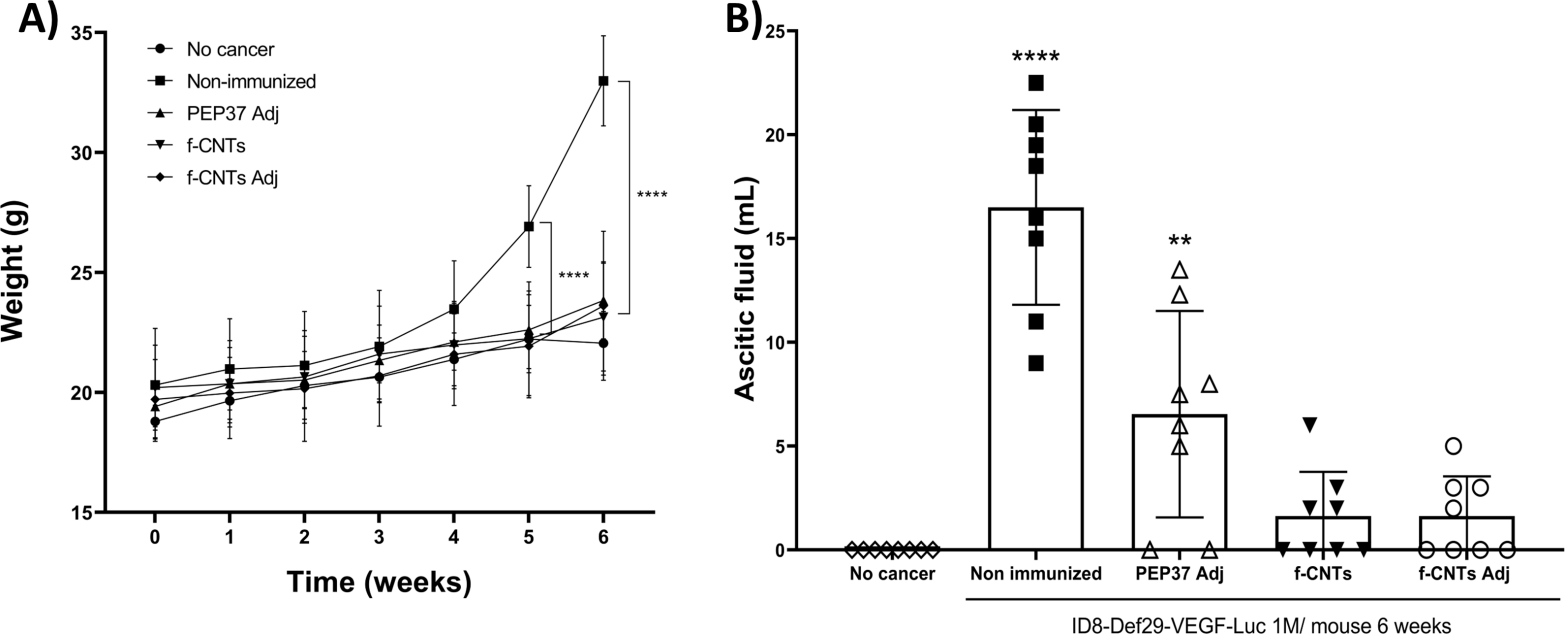
*f-*CNTs immunization delays disease progression and reduces ascitic fluid accumulation. **(A)** weekly body weights of the mice from the different groups over 6 weeks post-inoculation. **(B)** volume of ascitic fluid in mice from the different groups. Each bar shows the mean ± S.D. (each point represents a mouse; n=8). Panel **(A)**, asterisks indicate significant differences *vs* the non-immunized group. Panel **(B)**, asterisks indicate significant differences *vs* the no cancer group. **p < 0.01, **** p<0.0001 ANOVA and Tukey’s multiple comparison test. 1 M: 1 million inoculated ID8DVLuc cells.

To evaluate the association between immunization and disease progression, we used an immunocompetent ID8DVLuc model. In this model, disease progression is characterized by the development of disseminated peritoneal implants rather than a single measurable solid tumor mass. Therefore, weekly increases in body weight were attributed to the accumulation of ascitic fluid (**Figure 7A**).

Non-immunized mice accumulated large amounts of ascitic fluid, resulting in a significant increase in body weight at week 5, which became more pronounced by week 6 (**Figures 7A, B**). In contrast, compared with the no cancer group, all the immunized groups—regardless of the antigen or adjuvant used—showed no significant increase in body weight.

Similarly, the volume of ascitic fluid was significantly lower in all immunized groups than in the non-immunized group. Although the group immunized with *f-*CNTs plus adjuvant had the lowest ascites volume (**Figure 7B**), no statistically significant differences were observed among the immunized groups.

Overall, these findings indicate that immunization with *f-*CNTs is associated with delayed disease progression and reduced ascites accumulation in this model.

In addition, we conducted an independent experiment to evaluate overall survival through the follow-up of disease progression via *in vivo* bioluminescence and body weight data.

**Figure 8:**
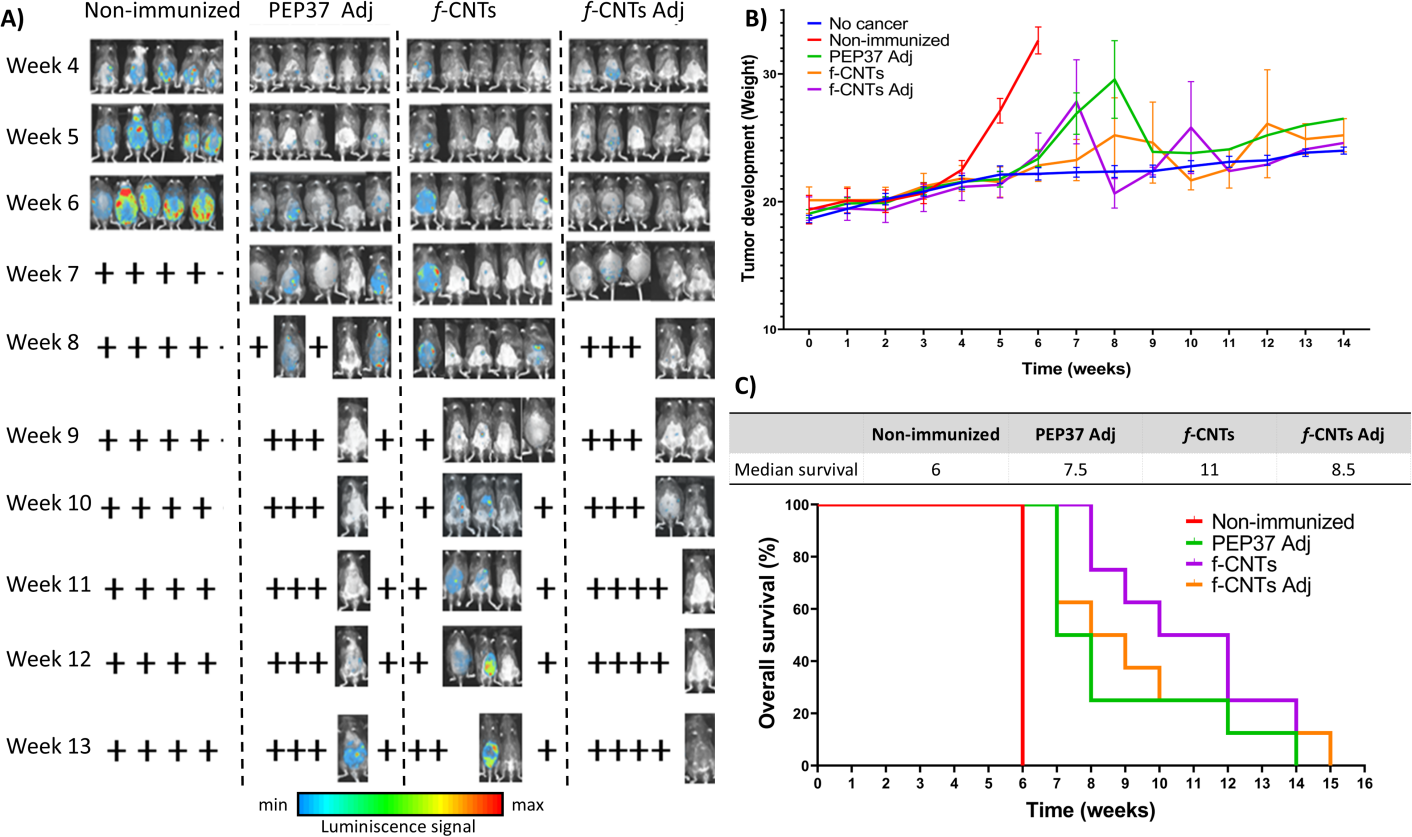
*f-*CNTs immunization prolongs overall survival in the ID8DVLuc ovarian cancer model. **(A)** disease progression was followed by bioluminescence, with images obtained each week; for practical purposes, we show weeks 4 to 13. **(B)** disease progression followed by weight increase caused by ascitic fluid accumulation. Each line represents a group (n=5). **(C)** overall survival curves. At the top, the mean overall survival in weeks for each group is displayed, and each line represents a group (two independent experiments, n=8 mice). Survival curves were compared using the log-rank (Mantel–Cox) test, which showed significant differences among groups (χ² = 32.80, df = 3, p < 0.0001). A significant trend was also observed (χ² = 11.45, df = 1, p = 0.0007). All groups showed significant differences, p < 0.0001 *vs* the non-immunized group. PEP37 Adj *vs*. *f*-CNTs p = 0.0436. PEP37 Adj *vs*. *f*-CNTs Adj p = 0.5665. *f*-CNTs *vs*. *f*-CNTs Adj p = 0.3727.

Non-immunized mice presented a stronger bioluminescence signal (**Figure 8A**) because the *Luc* gene was expressed in ID8DVLuc cells, which correlated with the weight increase in this group (**Figure 8B**) and an overall survival rate of no more than 6 weeks (**Figure 8C**).

In the PEP37 Adj-immunized mice, we observed an increase in the overall survival rate of 7.5 weeks on average; nevertheless, at week 9, most of the mice died because of ascitic fluid accumulation (**Figure 8**, PEP37 Adj). The results of the *f-*CNT plus adjuvant-immunized mouse group were similar to those of the PEP37 Adj-immunized group, with an overall survival rate of 8.5 weeks.

On the other hand, the mice immunized with *f-*CNTs without adjuvant had the best response, with a significant increase in overall survival (11 weeks). Even the mice that developed ascitic fluid showed weaker signals because of less disease progression (**Figures 8A, B**).

These results suggest that, compared with PEP37 Adj alone, bioconjugating PEP37 to CNTs induced an adequate immune response, as evidenced by an increase in overall survival in the ovarian cancer model (from 7.5–11 weeks). Second, the use of TiterMax as an adjuvant in the *f-*CNT plus adjuvant group, contrary to expectations, decreased overall survival compared with that in the *f-*CNT without adjuvant group (from 11 to 8.5 weeks), which could be related to the function of the adjuvant changing the response to a Th2 subpopulation or mixed Th1–Th2 population (**Figure 8C**). Third, these findings indicate that compared with unconjugated peptide immunization, immunization with *f-*CNTs is associated with prolonged survival in this ovarian cancer model.

### 3.7 Short-term histological assessment does not reveal overt tissue alterations.

Histopathological evaluation of major organs from all immunized groups revealed preserved tissue architecture with no detectable inflammatory infiltrates, necrosis, or structural abnormalities when compared with non-immunized controls (**Supplementary Figure 3**). This absence of overt morphological alterations was consistently observed in liver, heart, lung, and kidney tissues, supporting the lack of detectable systemic toxicity under the experimental conditions used.

Importantly, animals immunized with PEP37 Adj alone also exhibited normal tissue morphology, indicating that neither the peptide nor the nanoplatform-containing formulations induced evident short-term histological damage. Together with our previous *in vitro* cytotoxicity analysis (21), these findings support the short-term tolerability and preliminary safety profile of *f*-CNT-based immunization, at least within the tissues and time frame evaluated in this study.

## 4 Discussion

Fucosyltransferase 4 (FUT4) is widely reported to be overexpressed in multiple malignancies and to contribute to tumor progression and immune evasion mechanisms (13, 17, 18, 20). However, its immunogenic potential as a source of tumor-associated epitopes has not been previously explored in ovarian cancer models. In the present study, we evaluated a multiepitope peptide derived from murine FUT4 (PEP37), previously designed using bioinformatics and bioconjugated to carbon nanotubes (*f-*CNTs) (21,) and assessed its immunological and therapeutic effects in an ID8DVLuc ovarian cancer model.

First, we confirmed that FUT4 is overexpressed in tumor cells isolated from ascitic fluid in the ID8DVLuc model at six weeks post-inoculation. Increased expression was consistently observed by flow cytometry and immunofluorescence. Although the number of biological replicates was limited, the concordant results across independent techniques support the robustness of these observations. These findings suggest that FUT4 may be a relevant antigen source in this model, which is consistent with previous reports identifying overexpressed proteins as potential tumor-associated antigens (28, 29). Importantly, although the present work focused on the therapeutic evaluation of the nanovaccine in an ascites-driven murine model, independent ongoing analyses in human ovarian tumor tissues from our group further support the clinical relevance of FUT4 as a target in solid lesions across multiple histological subtypes, while showing minimal signal in non-cancerous ovarian tissue. Together, these observations reinforce the rationale for FUT4-directed immunotherapy as a potentially selective strategy against transformed ovarian tissue. Effective cancer immunotherapy must induce a robust immune response at the cellular level (through stimulation of cytotoxic T lymphocytes) or at the humoral level (through antibody production). Regardless of the response, recognition, internalization, processing, and presentation of antigens by APCs to T lymphocytes is needed. An essential factor in the design of cancer immunotherapy is that most antigens require additional molecules that act as adjuvants for their complete processing, thus improving their stability and antigenicity (10). In this case, we propose the use of CNTs as stimulatory particles to elicit an effective immune response to cancer antigens. Although we did not expect an increase in antibody titers, humoral responses were also enhanced following *f*-CNT immunization. Strong IgG and IgM responses were observed even in the absence of additional adjuvants, supporting the immunostimulatory properties of the CNT-based platforms. Future studies should define the precise FUT4-derived epitopes recognized by the *f*-CNTs-induced polyclonal antibodies, as this may help explain the functional contribution of humoral immunity to tumor control and potential antigen-spreading mechanisms. The addition of an external adjuvant increased antibody titers, but this immunization protocol did not improve survival and was associated with immune profiles suggestive of Th2 skewing or at least a mixed Th1/Th2 response.

In contrast, *f-*CNTs alone were associated with transcriptional signatures consistent with Th1 polarization and increased cytotoxic responses. These findings suggest that CNTs may act not only as antigen carriers but also as immunomodulatory platforms capable of influencing T-cell polarization. With respect to the cellular response, the transcriptional changes are consistent with enhanced antigen processing and MHC-I–associated presentation pathways, such as *H2-D1*, *H2-K1*, *B2m*, and *Cd74.* These genes are relevant because they are involved in antigen presentation in MH C class I, and the bioconjugated peptides are designed specifically for the H2-D and H2-K haplotypes. In addition, in this last pathway, CD74 is critical because the CD74-dependent MHC class I cross-presentation pathway in DCs plays a key role in the generation of MHC class I-restricted, cytolytic T lymphocytes (CTLs) in response to cell-associated antigens (30).

Functionally, immunization with *f-*CNTs was associated with reduced ascites accumulation and prolonged overall survival compared with those of nonimmunized mice and mice immunized with unconjugated peptide. Because the ID8DVLuc model primarily develops disseminated peritoneal implants rather than a single measurable solid mass, ascites volume and body weight are typically used as surrogate indicators of disease progression. While these measures do not fully capture total tumor burden, the consistent reduction in ascites combined with survival extension suggests a biologically meaningful effect of *f*-CNT immunization in this aggressive VEGF-driven model (31).

Ascitic accumulation in ovarian cancer has been linked to the predominance of immunosuppressive M2 macrophages. In intraperitoneal ID8 models, inhibition of M2 macrophages reduces ascitic fluid and increases CD8+ T-cell infiltration (32). In agreement with these reports, immunized mice—particularly those receiving *f-*CNTs—exhibited a shift from M2 to M1 macrophage profiles, accompanied by increased T-cell infiltration. These findings suggest that *f*-CNT immunization remodels the tumor microenvironment toward a more proinflammatory and potentially antitumoral state. In addition, these results are consistent with our previously published *in vitro* findings from our laboratory, demonstrating that these *f-*CNTs modulate macrophage polarization by promoting a shift from the M2 to the M1 phenotype. In those studies, we showed that *f-*CNT treatment enhanced the expression of proinflammatory markers while reducing the expression of M2-associated markers, supporting the functional reprogramming of macrophages toward an antitumoral phenotype (21).

Transcriptomic analysis further supported this immune remodeling. Although RNA-seq was performed on pooled samples and therefore should be interpreted as hypothesis-generating, the observed transcriptional changes were consistent with enhanced antigen processing and MHC-I–associated presentation pathways, including upregulation of H2-D1, H2-K1, B2m, and Cd74. Increased expression of genes associated with CD8+ T-cell activation (Cd8b1, Prf1, and Gzma) and Th1 polarization (Stat4 and Tbx21) was also detected. These findings were subsequently supported by flow cytometry and functional cytotoxicity assays, indicating that CD8+ T cells from *f*-CNT–immunized mice were capable of killing ID8DVLuc tumor cells *ex vivo*. The enrichment of CD8^+^ T cells observed after vaccination is consistent with the original multiepitope design of PEP37, which includes dominant H2-Db/H2-Kb-restricted motifs previously predicted for efficient MHC-I presentation (21). Although the present study suggests a localized cytotoxic immune remodeling within the ascitic tumor-associated compartment, an important next step will be to determine whether this spatial immune polarization is similarly established within solid metastatic peritoneal nodules. Comparative immune profiling of ascitic fluid and solid lesions will be particularly relevant to define whether the CD8^+^ enrichment, T-cell immune balance shift, and immune pathways identified in the current study are conserved across distinct ovarian cancer microenvironmental niches, thereby further strengthening the translational relevance of the nanovaccine platform.

The upregulation of Alox5 in *f*-CNT–immunized mice is noteworthy, as previous studies have shown that multiwalled CNTs can induce M1 polarization in murine macrophages through Alox5-dependent pathways (33). These results suggest a possible pathway for M1 polarization induction in *f*-CNT-immunized mice. Although mechanistic confirmation was not performed here, these observations suggest a potential pathway through which CNTs contribute to macrophage reprogramming.

The use of CNTs as vaccine platforms has been explored in infectious disease and cancer models. CNT-based conjugates have been shown to enhance antigen-specific IgG responses, promote dendritic cell uptake, and increase CD8+ T-cell activation in multiple preclinical settings (34–39). Our findings extend this body of work by demonstrating that FUT4-derived peptide bioconjugation to CNTs can modulate both innate and adaptive immune compartments in an aggressive ovarian cancer model.

The therapeutic benefit observed is unlikely to be driven by a single immune mechanism but rather reflects the coordinated contribution of multiple immune axes. In addition to cytotoxic T lymphocyte (CTL) responses, our findings indicate that humoral immunity, as evidenced by the antibody responses (**Figure 6**), and innate immune components, including macrophage polarization and functional activation, also contribute to the overall antitumor effect. The interplay among these mechanisms may enhance efficacy by promoting antigen recognition, facilitating effector cell recruitment, and sustaining immune activation within the tumor microenvironment. Collectively, these results support a model in which CTL, humoral, and innate immune responses act in a complementary and potentially synergistic manner to drive therapeutic outcomes.

The survival analyses demonstrate that immunization significantly impacts survival outcomes, as evidenced by the log-rank (Mantel–Cox) analysis showing robust differences among groups. Notably, all immunized groups exhibited a significant survival advantage compared with the non-immunized control, highlighting the overall efficacy of the immunization strategies. When comparing treatment modalities, the *f*-CNTs group showed a modest but statistically significant improvement over the PEP37 Adj group, suggesting a potential enhancement in antitumor efficacy associated with the nanomaterial-based platform. In contrast, no significant differences were observed between PEP37 Adj and *f*-CNTs Adj, nor between *f*-CNTs alone and *f*-CNTs Adj, indicating that the addition of adjuvant may not further improve survival outcomes in the context of the *f*-CNT formulation.

Together, these results suggest that while both immunization approaches confer protective effects, *f*-CNTs may intrinsically promote a more effective antitumor response, potentially through enhanced antigen delivery and immune activation. The lack of additive benefit from the adjuvant in the *f*-CNTs group further supports the notion that the nanomaterial itself may possess inherent immunostimulatory properties, consistent with its observed impact on immune cell activation and tumor-associated immune modulation.

Preliminary safety observations are particularly relevant considering concerns regarding FUT4-associated glycosylation in healthy tissues (**Supplementary Figure 3**). FUT4 is involved in the synthesis of fucosylated glycans, including Lewis antigens, which are not exclusively tumor-specific and can also be expressed at basal levels in normal epithelial and hematopoietic cells. This raises the possibility of off-target effects when targeting FUT4-related epitopes, particularly in tissues where fucosylation contributes to physiological processes such as cell adhesion, leukocyte trafficking, and immune homeostasis.

Importantly, our data did not reveal overt signs of toxicity or tissue damage under the evaluated conditions, suggesting that the targeting strategy may preferentially affect tumor-associated contexts where FUT4 expression or glycosylation patterns are dysregulated. However, the assessment of FUT4 expression in healthy tissues presents several limitations. Basal expression levels may be low and highly context-dependent, varying across tissue types and physiological states, which complicates direct comparisons. Collectively, these results provide preclinical proof-of-concept that CNT-based delivery of tumor-associated epitopes can reshape the tumor microenvironment and enhance antitumor immunity. Nevertheless, important limitations should be acknowledged, including small group sizes, reliance on surrogate tumor burden measures, and the exploratory nature of pooled RNA-seq analysis. Future studies incorporating larger cohorts, direct tumor implant quantification, and antigen-specific T-cell assays will be needed to further validate these findings and define the therapeutic potential of this platform. Finally, long-term immune memory, antigen spreading, and lesion-level mechanistic analyses remain important areas for future investigation and are now acknowledged as key directions to further define the durability and mechanisms of the antitumor response.

## 5 Conclusions

This study provides preclinical evidence that FUT4-derived peptides bioconjugated to carbon nanotubes can modulate the immune microenvironment in an ovarian cancer mouse model. FUT4 was found to be overexpressed in the tumor microenvironment of ID8DVLuc tumors, supporting its relevance as a source of tumor-associated epitopes.

Immunization with *f-*CNTs was associated with delayed disease manifestations, reduced ascitic fluid accumulation, and improved overall survival, together with immune remodeling characterized by M1 macrophage polarization, enhanced cytotoxic CD8^+^ T-cell activity, and transcriptional signatures consistent with Th1- and Th9-associated responses. Transcriptomic analyses, supported by immunophenotyping and functional assays, suggest the activation of antigen-processing and MHC class I–associated pathways following *f*-CNT immunization.

Although additional studies are needed to confirm antigen specificity and further define the underlying mechanism, these findings support the potential of carbon nanotube–based platforms as immunomodulatory carriers capable of enhancing peptide-driven antitumor immune responses in aggressive ovarian cancer models.

## Data Availability

The datasets presented in this study can be found in online repositories. The names of the repository/repositories and accession number(s) can be found below: https://www.ncbi.nlm.nih.gov/geo/, GSE299284.
