# Supplementary material for: Fucosyltransferase 4-derived peptide bioconjugates on carbon nanotubes enhance antitumor immunity in an ovarian cancer mouse model
Source: Front Immunol. 2026 May 19;17:1821727. doi: 10.3389/fimmu.2026.1821727 (PMC13226612; doi:10.3389/fimmu.2026.1821727)
Supplement: Supplementary file 1 [file Supplementaryfile1.docx]

**Supplementary material.**


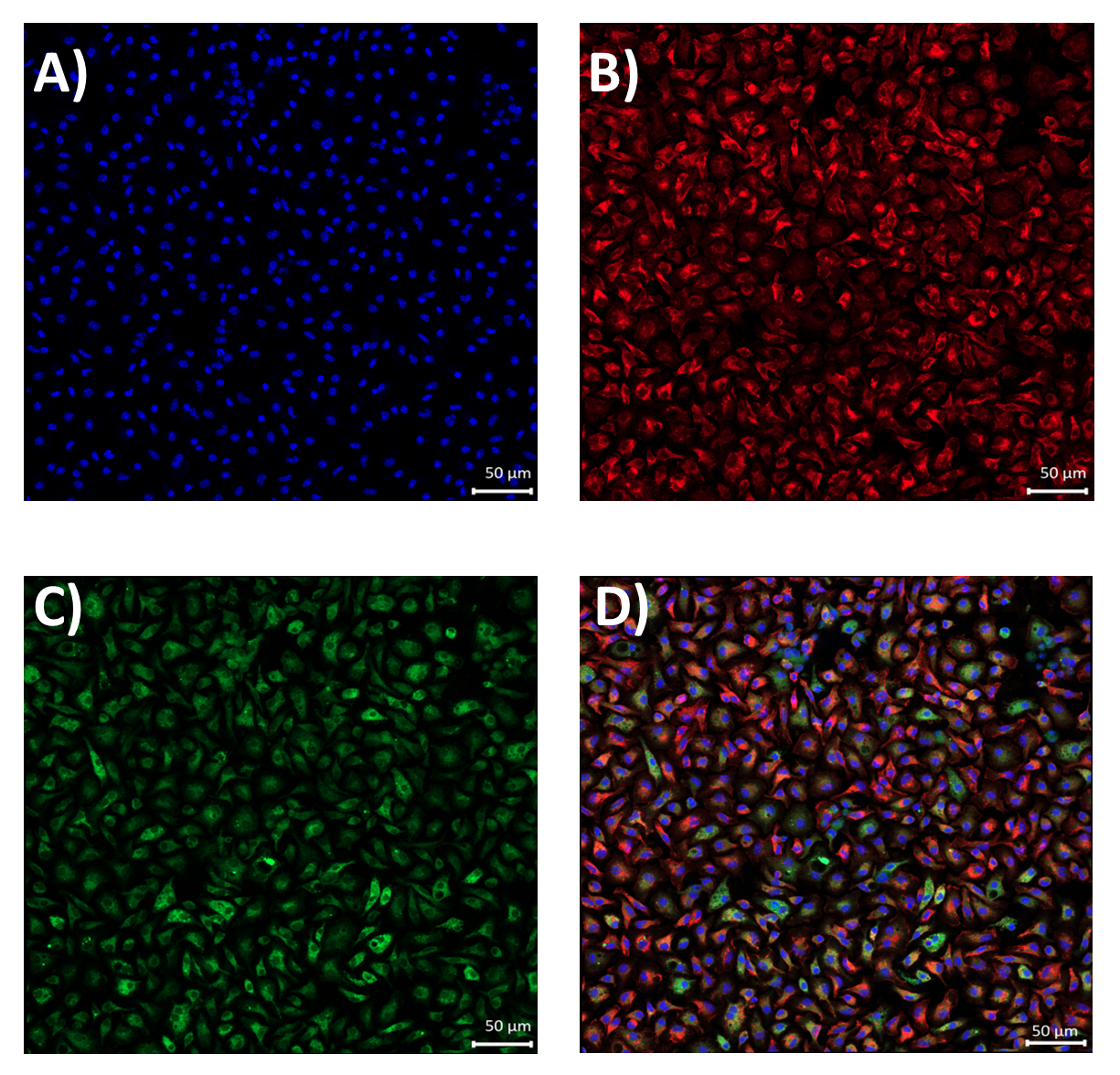


**Supplementary Figure 1.** Tiles scan 10X. ID8DVLuc cells identified in ascites by GFP expression. **A)** Blue, nuclei. **B)** Red, FUT4-AF647; **C)** Green, ID8DVLuc-GFP. **D)** Merge. All the cells displayed were both GFP- and FUT4-positive.


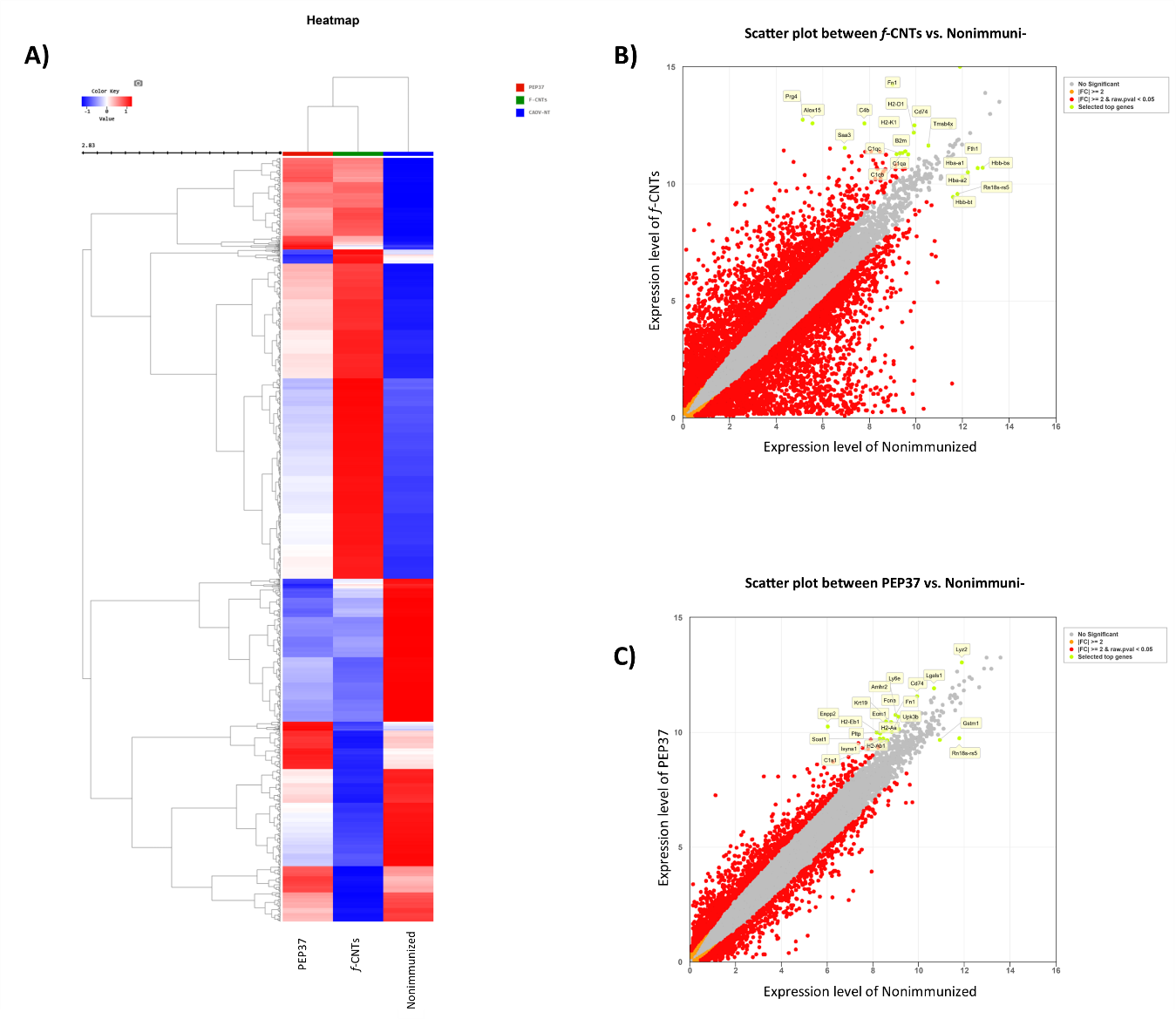


**Supplementary Figure 2. Genes differentially expressed between immunized and nonimmunized mice.** The nonimmunized profile was compared to that of PEP37 or *f-CNT*. **A)** Differential expression profile of the three groups, **B)** Scatter plot of DEGs in PEP37, and **C)** in *f-CNTs*.

**
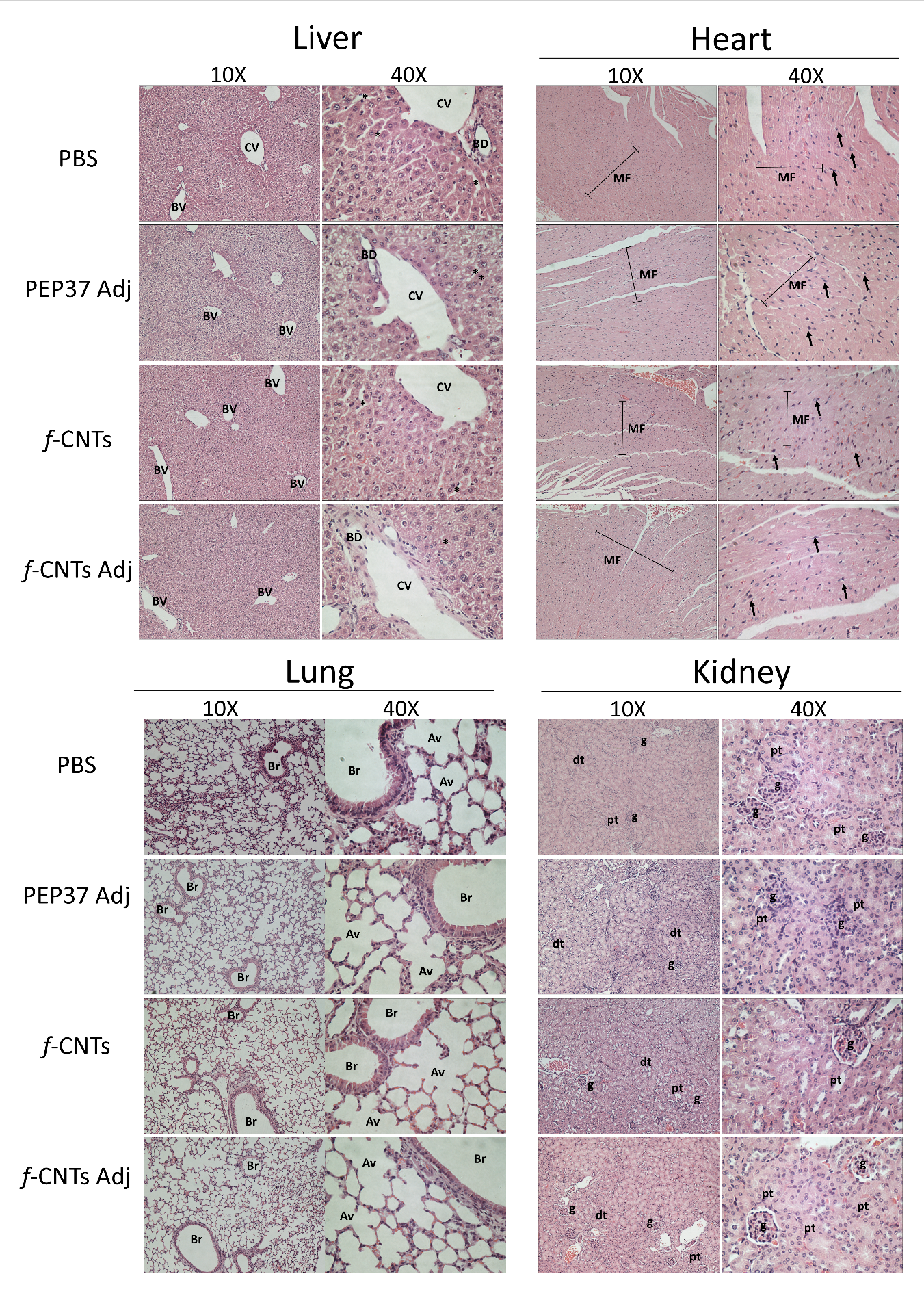
Supplementary Figure 3. Histopathological safety assessment of major organs following immunization.** Representative H&E-stained sections of liver, heart, lung, and kidney collected from mice treated with PBS, PEP37 Adj, *f*-CNTs, or *f*-CNTs adj. Low-magnification (10×) images show the overall tissue architecture, and the regions displayed at higher magnification (40×). No evident histopathological alterations, inflammatory infiltrates, necrotic areas, or structural abnormalities were observed in any organ across treatment groups, supporting the absence of overt systemic toxicity. CV, central vein; BV, blood vessel; BD, bile duct; MF, muscle fiber; arrows, nuclei; Br, bronchioles; Av, alveoli; g, glomeruli; dt, distal tubule; pt, proximal tubule


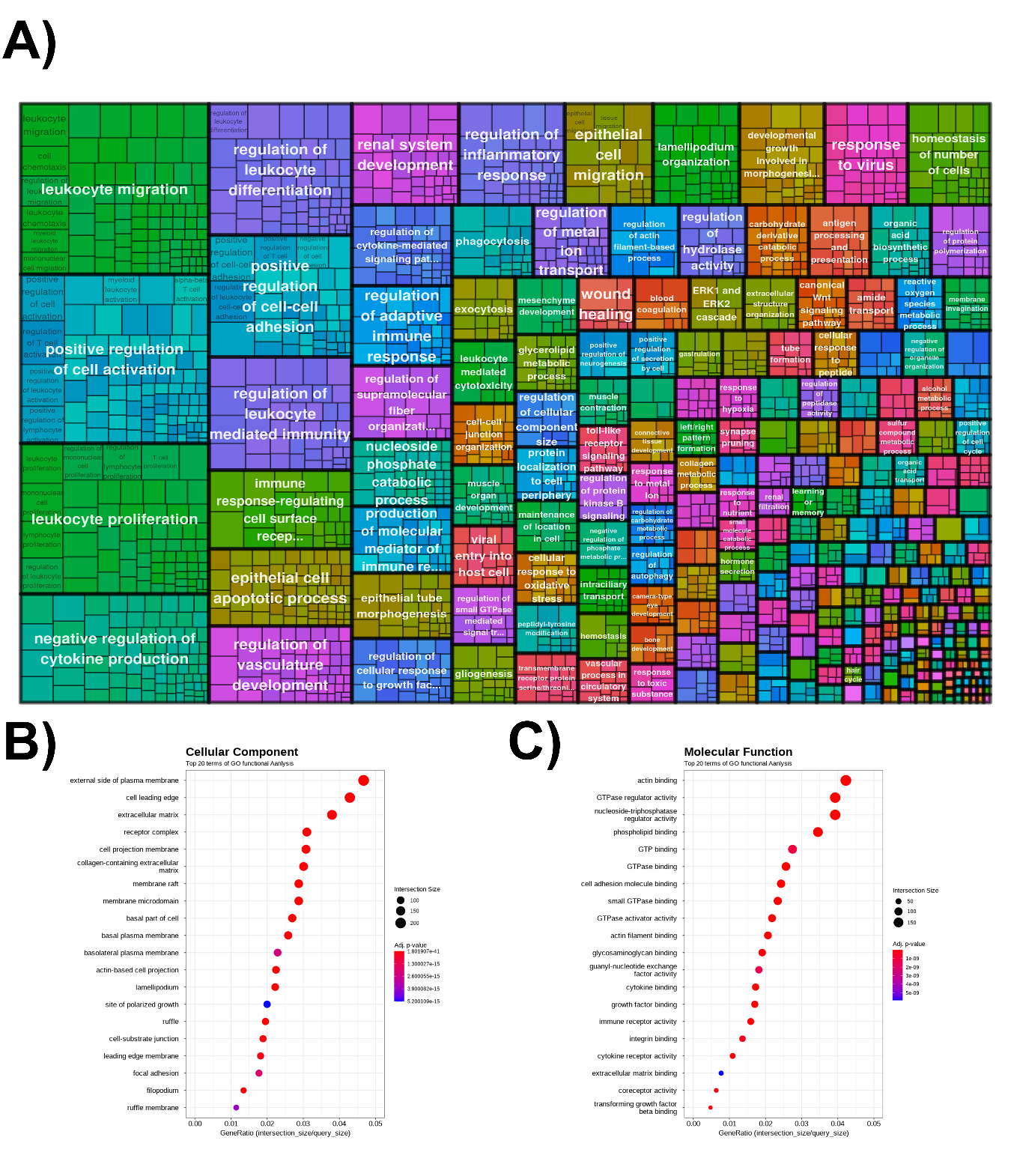


**Supplementary Figure 4. Gene ontology analysis of ascitic fluid cells from *f*-CNT-immunized mice.** **A)** Treemap plot. Treemaps are space-filling visualizations of hierarchical structures. The terms are grouped (colored) based on their parent, and the space used by the term is proportional to the score. **B)** Top 20 terms from the GO functional analysis of the CC category. **C)** Top 20 terms of GO functional analysis from the molecular function category.


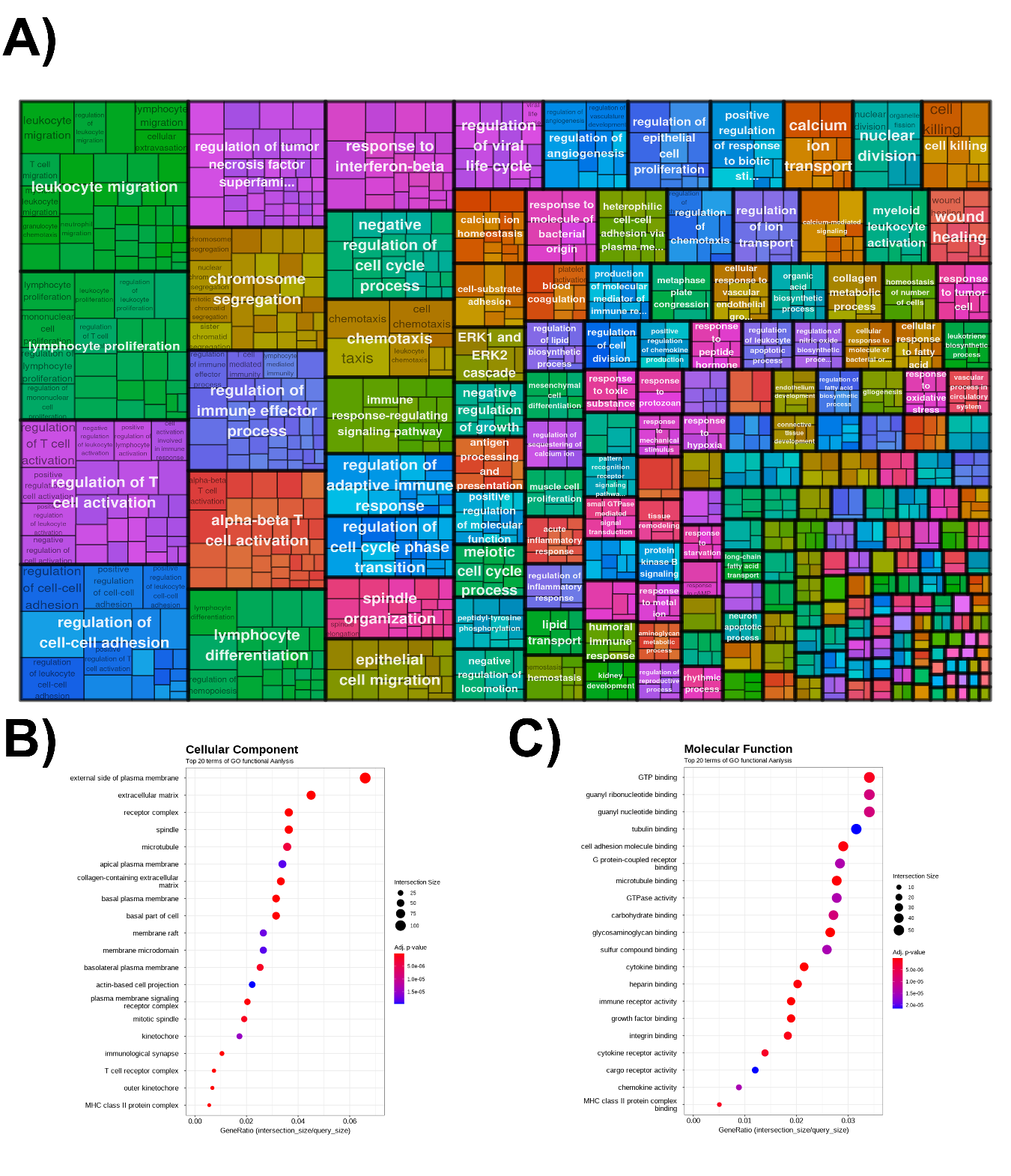


**Supplementary Figure 5. Gene ontology analysis of ascitic fluid cells from PEP37-immunized mice.** **A)** Treemap plot. Treemaps are space-filling visualizations of hierarchical structures. The terms are grouped (colored) based on their parent, and the space used by the term is proportional to the score. **B)** Top 20 terms from the GO functional analysis of the CC category. **C)** Top 20 terms of GO functional analysis from the molecular function category.


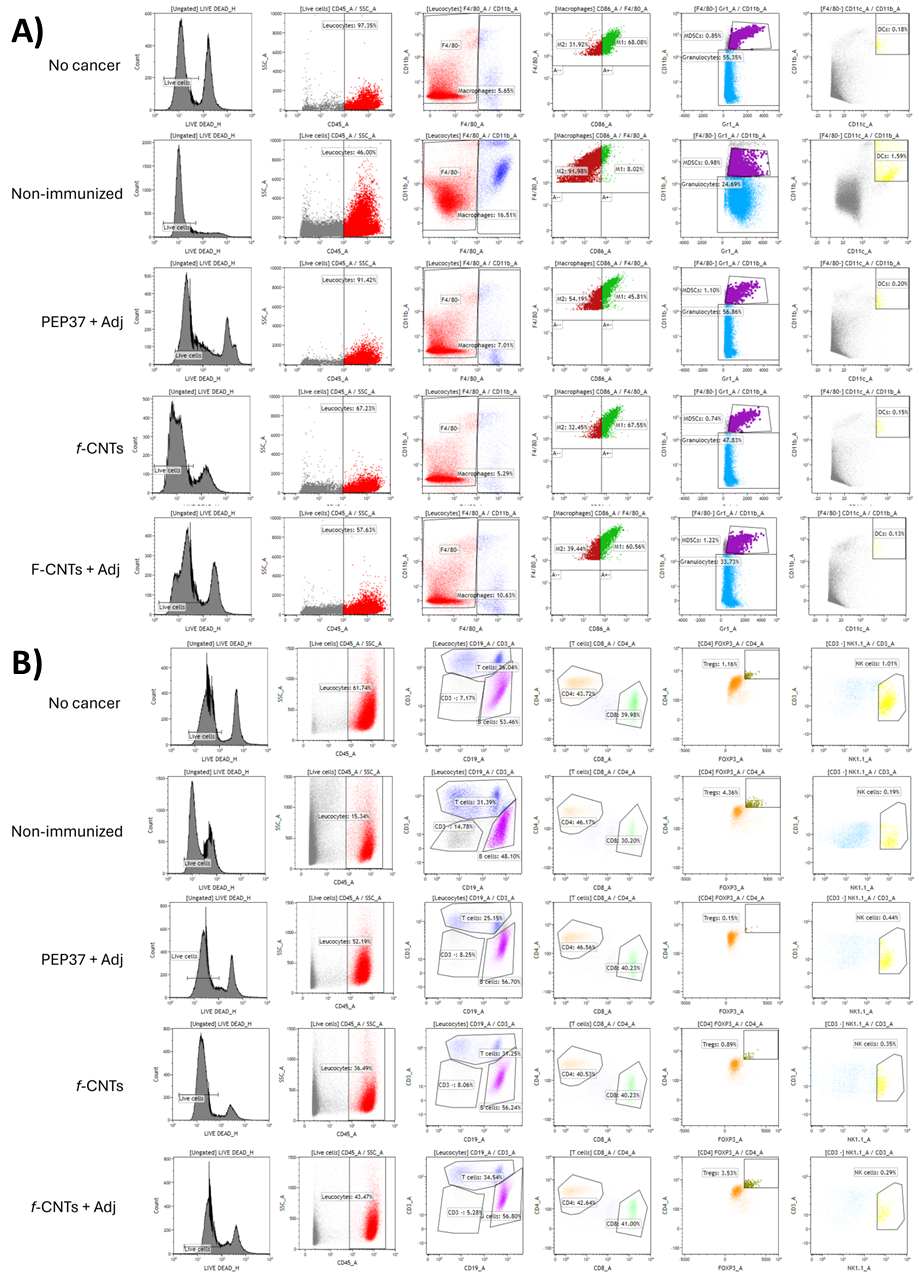


**Supplementary Figure 6. Representative flow cytometry gating strategy for myeloid and lymphoid populations across experimental groups. (A)** Myeloid compartment. Representative gating strategy used to identify myeloid cell populations. Briefly, live cells were first selected using a viability dye, followed by gating on CD45⁺ leukocytes. Within this population, macrophages were defined as CD11b⁺F4/80⁺ cells. Macrophage polarization was assessed based on CD86 expression. Granulocytes and myeloid-derived suppressor cells (MDSCs) were identified within the CD11b⁺ compartment using Gr-1 expression. Dendritic cells (DCs) were defined as CD11c⁺ cells within the CD11b⁺ fraction. (B) Lymphoid compartment. Representative gating strategy for lymphoid populations. After exclusion of dead cells and selection of CD45⁺ leukocytes, T and B cells were identified based on CD3 and CD19 expression, respectively. T cells were further subdivided into CD4⁺ and CD8⁺ subsets. Regulatory T cells (Tregs) were defined as CD4⁺FOXP3⁺ cells. Natural killer (NK) cells were identified as CD3⁻NK1.1⁺ cells. Representative plots from each experimental group (No cancer, Non-immunized, PEP37 Adj, *f*-CNTs, and *f*-CNTs Adj) are shown.

**Supplementary Table 1.** Flow cytometry antibodies used for immune profiling and functional analyses

| Biomarker / Cell population detected | Antibody (fluorophore) | Clone | Supplier | Catalog number |
| --- | --- | --- | --- | --- |
| Total leukocytes | CD45-APC | 30-F11 | BioLegend | 103112 |
| Total T cells | CD3-V500 | 500AZ | BD Biosciences | 560771 |
| Cytotoxic T cells (CD8⁺) | CD8b-PE/Dazzle 594 | YTS156.7.7 | BioLegend | 126621 |
| Helper T cells (CD4⁺) | CD4-APC-Cy7 | GK1.5 | BD Biosciences | 552051 |
| B cells | CD19-PE | eBio1D3 | eBioscience | 12-0193-82 |
| NK cells | NK1.1-BB700 | PK136 | BD Biosciences | 566502 |
| Regulatory T cells (Tregs) | FOXP3-AF700 | MF-14 | BioLegend | 126421 |
| Macrophages | F4/80-PE | BM8 | BioLegend | 120309 |
| Neutrophils / granulocytic cells | Ly6G (Gr-1)-AF700 | RB6-8C5 | BioLegend | 108421 |
| Myeloid cells / macrophage gating | CD11b-PE-Cy7 | M1/70 | BioLegend | 101215 |
| M1-like macrophage activation | CD86-FITC | GL1 | eBioscience | 11086282 |
| Dendritic cells | CD11c-PE-Cy5 | N418 | eBioscience | 15011481 |
| CD8⁺ T-cell effector function (IFNγ) | IFNγ-BV650 | XMG1.2 | BD Biosciences | 563854 |
| CD8⁺ T-cell effector function (TNF) | TNF-V450 | MP6-XT22 | BD Biosciences | 560655 |
| Cytotoxic granule marker | Granzyme B-PE | QA16A02 | BioLegend | 372208 |

**Abbreviations:** IFNγ, interferon gamma; TNF, tumor necrosis factor alpha.

**Supplementary Table 2**. Comparative analysis of CD4⁺ and CD8⁺ T-cell frequencies and CD4/CD8 ratio in systemic (spleen) and tumor-associated (ascitic fluid) compartments.

| Group | Total, CD3+, CD4+ cells (%) | Total, CD3+, CD8+ cells (%) | CD4/CD8 Ratio |
| --- | --- | --- | --- |
| Spleen | | | |
| No cancer | 45.0 | 30.7 | 1.46 |
| Non immunized | 41.1 | 29.1 | 1.41 |
| PEP37 Adj | 46.5 | 33.0 | 1.40 |
| *f*-CNTs | 42.6 | 34.4 | 1.23 |
| *f*-CNTs Adj | 40.0 | 33.2 | 1.20 |
| Ascitic fluid | | | |
| No cancer | 58.7 | 13.1 | 4.45 |
| Non immunized | 26.0 | 25.9 | 1.00 |
| PEP37 Adj | 30.9 | 32.5 | 0.95 |
| *f*-CNTs | 22.2 | 28.4 | 0.78 |
| *f*-CNTs Adj | 47.9 | 12.5 | 3.82 |
